# Head-to-head comparison of [^177^Lu]Lu-FAP-2286 and [^161^Tb]Tb-FAP-2286 efficacy in a PDAC mouse model

**DOI:** 10.1186/s13550-026-01372-5

**Published:** 2026-01-09

**Authors:** Circe D. van der Heide, Carolline M. Ntihabose, Mark Konijnenberg, Hanyue Ma, Debra Stuurman, Corrina de Ridder, Yann Seimbille, Michail C. Doukas, Erik de Blois, Simone U. Dalm

**Affiliations:** 1 Department of Radiology & Nuclear Medicine, Erasmus MC, Rotterdam, The Netherlands; 2 Department of Hospital Pharmacy, Erasmus MC, Rotterdam, The Netherlands; 3 Department of Experimental Urology, Erasmus MC, Rotterdam, The Netherlands; 4 Department of Pathology, Erasmus MC, Rotterdam, The Netherlands

**Keywords:** Fibroblast activation protein (FAP), Cancer-associated fibroblast (CAF), Targeted radionuclide therapy (TRT), Pancreatic ductal adenocarcinoma (PDAC), Terbium-161

## Abstract

**Background:**

Terbium-161 (Tb-161) emits internal conversion and Auger electrons, in addition to beta-minus radiation, which might be of added benefit for targeted radionuclide therapy (TRT) compared to Lutetium-177 (Lu-177). We extensively compared Lu-177 and Tb-161 for fibroblast activation protein (FAP)- TRT in a preclinical setting. To study this, FAP-2286 was labeled with Lu-177 and Tb-161 and characterized in vitro on FAP-expressing cells and ex vivo using patient tumor samples. Moreover, in vivo studies (i.e. biodistribution and efficacy) were performed using a clinically representative pancreatic ductal adenocarcinoma (PDAC) mouse model. Biodistribution was performed 1, 4, 24, and 48 h post injection of 5 MBq/500 pmol [^177^Lu]Lu-FAP-2286 or [^161^Tb]Tb-FAP-2286. Subsequently, animals were treated with 4 × 40 MBq/500 pmol [^177^Lu]Lu-FAP-2286 or [^161^Tb]Tb-FAP-2286 and with alternating doses of 2 × 40 MBq/500 pmol of each radiopharmaceutical.

**Results:**

No difference in [^177^Lu]Lu-FAP-2286 and [^161^Tb]Tb-FAP-2286 uptake was observed in the cell models. In vivo studies did not show a survival benefit of 4 × 40 MBq/500 pmol [^177^Lu]Lu-FAP-2286 or [^161^Tb]Tb-FAP-2286, while Kaplan-Meier analyses demonstrated a modest prolonged survival after tandem therapy in mice that first received [^177^Lu]Lu-FAP-2286 followed by [^161^Tb]Tb-FAP-2286. Dosimetry calculations based on autoradiography studies on patient tumor samples showed that even with lower binding, a higher absorbed dose to the tumor can be accomplished with [^161^Tb]Tb-FAP-2286.

**Conclusions:**

In our in vitro and in vivo studies, [^177^Lu]Lu-FAP-2286 and [^161^Tb]Tb-FAP-2286 demonstrated similar behavior. In the applied PDAC mouse model, FAP-TRT showed limited therapeutic efficacy, most likely due to the limited radiopharmaceutical uptake observed in the tumors. This hampered determination of a potential benefit of either radioisotope for FAP-TRT. Of note, a modest response was observed in the tandem therapy group that first received [^177^Lu]Lu-FAP-2286, followed by [^161^Tb]Tb-FAP-2286.

**Supplementary Information:**

The online version contains supplementary material available at 10.1186/s13550-026-01372-5.

## Background

Pancreatic ductal adenocarcinoma (PDAC) is an aggressive cancer type with a poor 5-year survival rate of less than 10% [1]. PDAC is typically treatment resistant, amongst others, due to the presence of a dense tumor stroma. The most abundant cellular component of this tumor stroma is the cancer-associated fibroblast (CAF), which has been associated with therapy resistance and suppression of immune cells [2]. CAFs are characterized by the expression of fibroblast activation protein (FAP), which is only scarcely expressed in healthy tissues [3]. This cancer specific and abundant expression of stromal FAP in PDAC, and other solid cancers, makes it an attractive biomarker for anti-cancer interventions, including targeted radionuclide therapy (TRT) [4, 5]. Currently, FAP-2286 is one of the most promising compounds for FAP-TRT, due to its relatively high tumor retention [6]. Accordingly, a large prospective clinical Phase I/II trial with [^68^Ga]Ga/[^177^Lu]Lu-FAP-2286 to determine the potential of FAP-targeted theranostics in PDAC and other cancer types is ongoing (NCT04939610).

At present, [^177^Lu]Lu-DOTATATE and [^177^Lu]Lu-PSMA-617 are clinically approved for TRT of somatostatin receptor subtype 2 (SSTR2)-expressing neuroendocrine tumors (NETs) and prostate specific membrane antigen (PSMA)-expressing metastatic castration-resistant prostate cancer (mCRPC), respectively [7, 8]. Unfortunately, [^177^Lu]Lu-DOTATATE and [^177^Lu]Lu-PSMA-617 treatment efficacy can be limited, and a subset of patients do not respond to treatment despite prominent radiopharmaceutical uptake in tumors on PET scans acquired using the same or a related SSTR2- or PSMA-targeting radiopharmaceutical, coupled to e.g. gallium-68, prior to treatment. To enhance therapeutic efficacy, cytotoxic radionuclides other than lutetium-177 (Lu-177) are being studied. Among them is terbium-161 (Tb-161), which has gained considerable interest recently [9]. The decay characteristics of Tb-161 are similar to that of Lu-177 (Lu-177: Eβ^−^_av_ = 133 keV, T_1/2_ = 6.65 days vs. Tb-161: Eβ^−^_av_ = 154 keV, T_1/2_ = 6.91), but besides a slightly higher β^−^-energy, Tb-161 emits a significant amount of internal conversion electrons (CEs) and Auger electrons (AEs) [10]. These CEs and AEs have a higher linear energy transfer (LET) than the β^−^ particles, and potentially have added therapeutic benefit, especially when in close proximity of the DNA [11]. Monte Carlo simulations have predicted an increased absorbed dose of Tb-161 over Lu-177 [12, 13], and in line with this, preclinical studies demonstrated improved efficacy of Tb-161 labeled PSMA- and SSTR2-targeting radiopharmaceuticals over the Lu-177 labeled counterparts [13, 14]. Based on these promising results, clinical trials evaluating ^161^Tb-labeled radiopharmaceuticals for NET and mCRPC treatment are ongoing [15] (e.g. NCT04833517, NCT05521412, NCT06343038, NCT05359146).

Tb-161 can potentially also be more beneficial than Lu-177 for FAP-TRT. Presumably, FAP-TRT causes cancer cell death by irradiating the cancer cells indirectly via the FAP-expressing CAFs. However, especially in stroma-dense tumors such as PDAC, CAF elimination could also be a successful treatment strategy to reduce treatment resistance and potentially improve efficacy of other anti-cancer therapies. It remains to be determined whether the additional short-range CEs and AEs emitted by Tb-161 can induce greater CAF and/or cancer cell death compared to Lu-177. Accordingly, we performed in vitro and in vivo studies to compare [^177^Lu]Lu-FAP-2286 and [^161^Tb]Tb-FAP-2286, aimed at evaluation of their efficacy in a clinically representative PDAC mouse model after both mono- and tandem TRT.

## Methods

### Radiolabeling and stability

FAP-2286 (MedChem Express, Monmouth Junction NJ, USA) was labeled with either non-carrier added [^177^Lu]LuCl_3_ (Lu-177, PI Medical, Raamsdonksveer, The Netherlands) or non-carrier added [^161^Tb]TbCl_3_ (Tb-161, TerThera BV, Breda, The Netherlands) in a total volume of 140 µL. The radiochemical purity (RCP) was determined by high-performance liquid chromatography (HPLC). Stability was determined by analyzing the RCP after 2 and 24 h, by incubating 2.5 MBq radiolabeled FAP-2286 in 200 µL phosphate buffered saline (PBS) (Gibco, Breda, The Netherlands) or 100 µL commercially available mouse serum (Invitrogen, Carlsbad CA, USA) at 37 °C. Details of the labeling and stability studies are described in the Supplementary Information.

### Cell culture

Human fibrosarcoma cells transduced to express human FAP (huFAP) (i.e. HT1080-huFAP), kindly provided by Prof. Uwe Haberkorn (Dept of Nuclear Medicine, University of Heidelberg, Heidelberg, Germany), and pancreatic stellate cells (i.e. PS-1), provided by Queen Mary University (London, United Kingdom), were cultured as previously described [16]. The 19TT-F human breast CAFs were obtained from Prof. John Martens (Dept of Medical Oncology, Erasmus MC, Rotterdam, The Netherlands) and were cultured in RPMI Glutamax© (Gibco). One day prior to in vitro assays, cells were seeded in 12-well plates (Sarstedt, Nümbrecht, Germany) to reach 80% confluence. For in vivo studies, a cell line-derived xenograft (CDX) model was established using the T110299 cell line, derived from a Ptf1a^WT/Cre^ Kras^WT/LSL−G12D^ P53^LSL − R172H/fl^ mouse that spontaneously grows primary PDAC [17]. T110299 cells, provided by University Hospital Essen (Essen, Germany), were cultured in DMEM Glutamax© containing pyruvate (Gibco). All cell culture media were supplemented with 10% fetal bovine serum (Gibco) and 100 UI/mL penicillin + 100 µg/mL streptomycin (Merck Life Science NV, Amsterdam, The Netherlands).

### In vitro competition binding and uptake

The half-maximal inhibitory concentration (IC50) of the radiopharmaceuticals was determined on HT1080-huFAP cells using 1 nM [^177^Lu]Lu-/[^161^Tb]Tb -FAP-2286, co-incubated with increasing concentrations (10^− 12^ to 10^− 6^ M) of the FAP-inhibitor UAMC-1110 (Biosynth Ltd, Berkshire, United Kingdom) for 45 min. To determine radiopharmaceutical uptake, HT1080-huFAP, PS-1, and 19TT-F cells were incubated with 1 nM [^177^Lu]Lu-/[^161^Tb]Tb-FAP-2286 for 5–180 min, + (blocked)/- (non-blocked) 1 mM UAMC-1110 to determine specificity of uptake, as previously described [16]. Radiopharmaceutical uptake measured in blocked conditions was subtracted from radiopharmaceutical uptake measured in non-blocked conditions resulting in values indicating FAP specific uptake only. The uptake is expressed as percentage added activity (%AA) per 200,000 cells.

### In vitro autoradiography

Radiopharmaceutical binding to fresh frozen 10 μm slices of T110299 CDXs, human PDAC, and breast cancer (BC) tissue (*n* = 3/group), mounted onto Superfrost Plus™ microscope slides (VWR, Leuven, Belgium), was assessed as previously described [18]. In short, tissue slices were incubated with incubation buffer (167 mM Tris-HCL, 5 mM MgCl_2,_ 1% BSA) containing 1 nM [^177^Lu]Lu-/[^161^Tb]Tb-FAP-2286, + (blocked)/- (non-blocked) 1 mM UAMC-1110 to determine specificity of binding. Tissues were exposed to super-resolution phosphor screens (Perkin Elmer, Drachten, The Netherlands) for at least 24 h. Uptake was quantified as digital light units (DLU)/mm^2^ and normalized to standards obtained by quantifying 1 µL drops of the incubation solution. Radiopharmaceutical binding quantified of blocked conditions was subtracted from radiopharmaceutical binding quantified of non-blocked conditions resulting in values indicating FAP specific binding only. The data are expressed as % AA.

### Animal model

For all in vivo studies, C57BL/6 male mice (Janvier, Le Genest-Saint-Isle, France) were subcutaneously inoculated with 50 µL HBSS (Gibco) containing 0.5 × 10^6^ T110299 cells, with a tumor take of 100%. Details on inoculation and tumor growth, animal welfare, and animal numbers are provided in the Supplementary Information (Table S1−2). In all studies, animals were monitored daily, and animal weight and tumor size was measured twice each week.

### Ex vivo biodistribution and ex vivo autoradiography

On day 11 after inoculation of T110299 cells (tumor sizes; Lu-177: 431 ± 173 mm^3^ and Tb-161: 315 ± 159 mm^3^ (Fig. S1)), animals received an intravenous injection (IV) in the tail vain of [^177^Lu]Lu-/[^161^Tb]Tb-FAP-2286 (5 MBq/500 pmol/200 µL), diluted in PBS containing 0.06 mg/mL Kolliphor^®^ HS 15 (Sigma Aldrich, Saint-Louis MS, USA) . At 1, 4, 24, and 48 h post injection (p.i.) (*n* = 5/group), blood was collected via orbital puncture under isoflurane/O_2_ anesthesia, followed by cervical dislocation and collection of tumor and organs of interest (i.e., pancreas, liver, GI-tract (stomach, small intestine, cecum, colon), kidneys, lungs, heart, salivary glands, muscle, femur bone). A subset of animals (*n* = 4/group) received an injection of radiolabeled FAP-2286 + 17.5 nM UAMC-1110 following biodistribution studies 4 h p.i. to determine specificity of uptake (i.e. blocked condition). The collected materials were weighed and measured in a γ-counter. Data are expressed as percentage injected dose per gram of tissue (% ID/g). Half of the tumor was snap frozen in liquid nitrogen, sectioned (10 μm), mounted on Starforst glass slides, and exposed to a super-resolution phosphor screen to determine radiopharmaceutical binding.

### In vivo efficacy of mono and tandem treatment

T110299 tumors were allowed to grow for seven days reaching a size of 89 ± 38 mm^3^. On day 7, 10, 14, and 17 after T110299 cell inoculation, mice received IV injections of 200 µL 40 MBq/500 pmol [^177^Lu]Lu-/[^161^Tb]Tb-FAP-2286 (*n* = 10/group), or vehicle solution (PBS + 0.06 mg/mL Kolliphor^®^ HS 15) (*n* = 8). An additional group of animals (*n* = 2–3/group) were sacrificed 1 h after each IV injection, and tumors were harvested to measure radiopharmaceutical uptake and to assess potential changes in FAP-expression or stroma-to-cancer cell ratios over time. Immunohistochemistry (IHC) was performed on the excised tumors and a pathologist (MCD) scored the tumor tissues blindly to determine the percentage of tumor cells and stroma/CAFs of the total tumor area. Details on the IHC are described in the Supplementary Information.

Next, tandem efficacy was determined in T110299 xenografted mice with an average tumor size of 219 ± 83 mm^3^ on day seven post inoculation. Mice received vehicle or 40 MBq/500 pmol radiopharmaceutical on day 8, 11, 15, and 18 post T110299 inoculation. Animals were treated with 2×[^177^Lu]Lu-FAP-2286 followed by 2×[^161^Tb]Tb-FAP-2286, or vice versa (*n* = 13/group), or vehicle containing PBS + 0.06 mg/mL Kolliphor^®^ HS 15 (*n* = 9) (Fig. S2).

### Dosimetry

The biodistribution data of [^177^Lu]Lu-FAP-2286 and [^161^Tb]Tb-FAP-2286 were used to characterize the organ and tumor time-activity curves. These curves were fitted with mono-exponential curves to enable integration over time, leading to Time Integrated Activity (concentration) Coefficients ([TIAC]). Absorbed doses to the mouse organs and tumor per injected activity (IA) were calculated according to the modified MIRD equation:


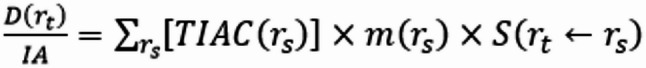


with *r*_*t*_ the target organ or tumor, *r*_*s*_ the source organ, *m(r*_*s*_*)* the mass of the source organ and *S(r*_*t*_←*r*_*s*_*)* the S-value. Both the organ masses and the S-values were taken from the RADAR 25 g mouse phantom [19]. The tumor absorbed doses were calculated by using the sphere S-values from the Olinda dosimetry software [20]. Calculations were used to estimate the treatment response, taking the radiosensitivity of the tumor cells [21], their doubling time, and the kinetics of FAP-2286 and its dosimetry when labeled to Lu-177 or Tb-161, into account.

Dosimetry in the PDAC and BC tissues was performed by inserting the autoradiography data as source distributions in a cylindrical tissue model set up in the Monte Carlo code MCNP (version 6.2). Autoradiography images were resampled to a pixel size of 85 μm and all data above 1800 DLU/mm^2^ were used as source in the middle of a cylindrical tissue model of 3 mm thickness. The energy absorption inside an 85 × 85 × 3000 µm^3^ voxel grid was tallied and transformed into S-values [in mGy/MBq.s]. The Tb-161 and Lu-177 beta-particle and low energy electron spectra of ICRP-107 were used. The number of histories used in the source distribution was set at 10 million to obtain statistical uncertainties below 5% in each voxel. Absorbed dose was subsequently calculated using ImageJ software (version 1.54 g) by drawing and measuring the whole tissue slice or the tumor area in accordance with the pathologists findings in the corresponding H&E slice.

### Statistics

Statistical analyses were performed using GraphPad Prism 9.0 (San Diego, California USA), and results were regarded statistically significant if *p* < 0.05. All experiments were performed at least in triplicate, unless stated otherwise. More detailed information is provided in the Supplementary Information.

## Results

### Stability in vitro

The stability of [^177^Lu]Lu-FAP-2286 and [^161^Tb]Tb-FAP-2286 in labeling solution was high, and there was no significant difference between the radiopharmaceuticals, i.e. 97.6 ± 0.6% vs. 95.2 ± 2.8% (*p* > 0.05), respectively, after 2 h incubation, and 93.4 ± 2.1% and 93.4 ± 2.6%, (*p* > 0.05), respectively, after 24 h incubation. In contrast, in mouse serum a decrease in stability was observed for both [^177^Lu]Lu-FAP-2286 and [^161^Tb]Tb-FAP-2286. A significant difference in stability between the radiopharmaceuticals was observed after 2 h, but not after 24 h of incubation (i.e. 70.0 ± 0.5% vs. 75.8 ± 1.0% *p* < 0.001; and 13.0 ± 0.5% vs. 12.2 ± 0.5% *p* > 0.05, respectively) (Table S3).

### In vitro competition binding and uptake

**Fig. 1:**
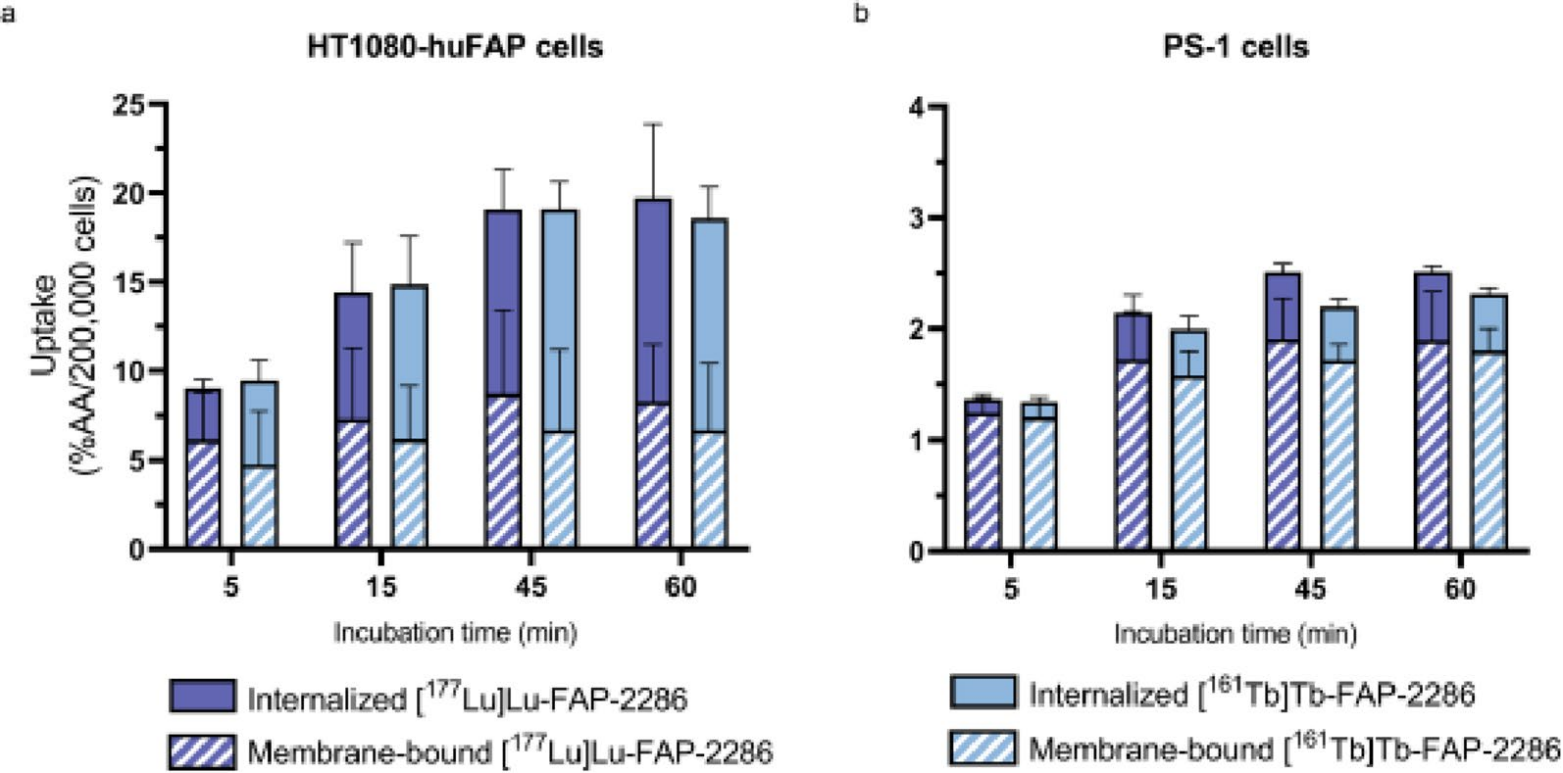
Membrane-bound and internalized [^177^Lu]Lu-FAP-2286 vs. [^161^Tb]Tb-FAP-2286 in (**a**) HT1080-huFAP cells (ns) and (**b**) PS-1 CAFs after 5–60 min of incubation (ns) (*n* = 3). *%AA = percentage added activity*,* ns = non-significant*

There were no significant differences in either the IC50 (i.e. Lu-177: 2.9 (95%CI 0.59–14.9) nM vs. Tb-161: 1.5 (95%CI 0.3–6.8) nM, *p* > 0.05, Fig. S3), or in the uptake of [^177^Lu]Lu-FAP-2286 and [^161^Tb]Tb-FAP-2286 evaluated in HT1080-huFAP and in PS-1 cells (*p* > 0.05) (Fig. 1). Moreover, radiopharmaceutical uptake was specific; no/little uptake was observed in blocked conditions. Radiopharmaceutical uptake occurred rapidly in both cell lines, but was 5–10 fold higher in HT1080-huFAP cells than in PS-1 cells. Moreover, radiopharmaceutical uptake was largely internalized by HT1080-huFAP cells, whereas in PS-1 cells it remained mainly membrane-bound. No differences in the internalization ratio was found between [^177^Lu]Lu-FAP-2286 and [^161^Tb]Tb-FAP-2286 in either cell line. Matching uptake of the two radiopharmaceuticals was further confirmed in 19TT-F cells (*p* > 0.05, Fig. S4).

### Ex vivo biodistribution

**Fig. 2:**
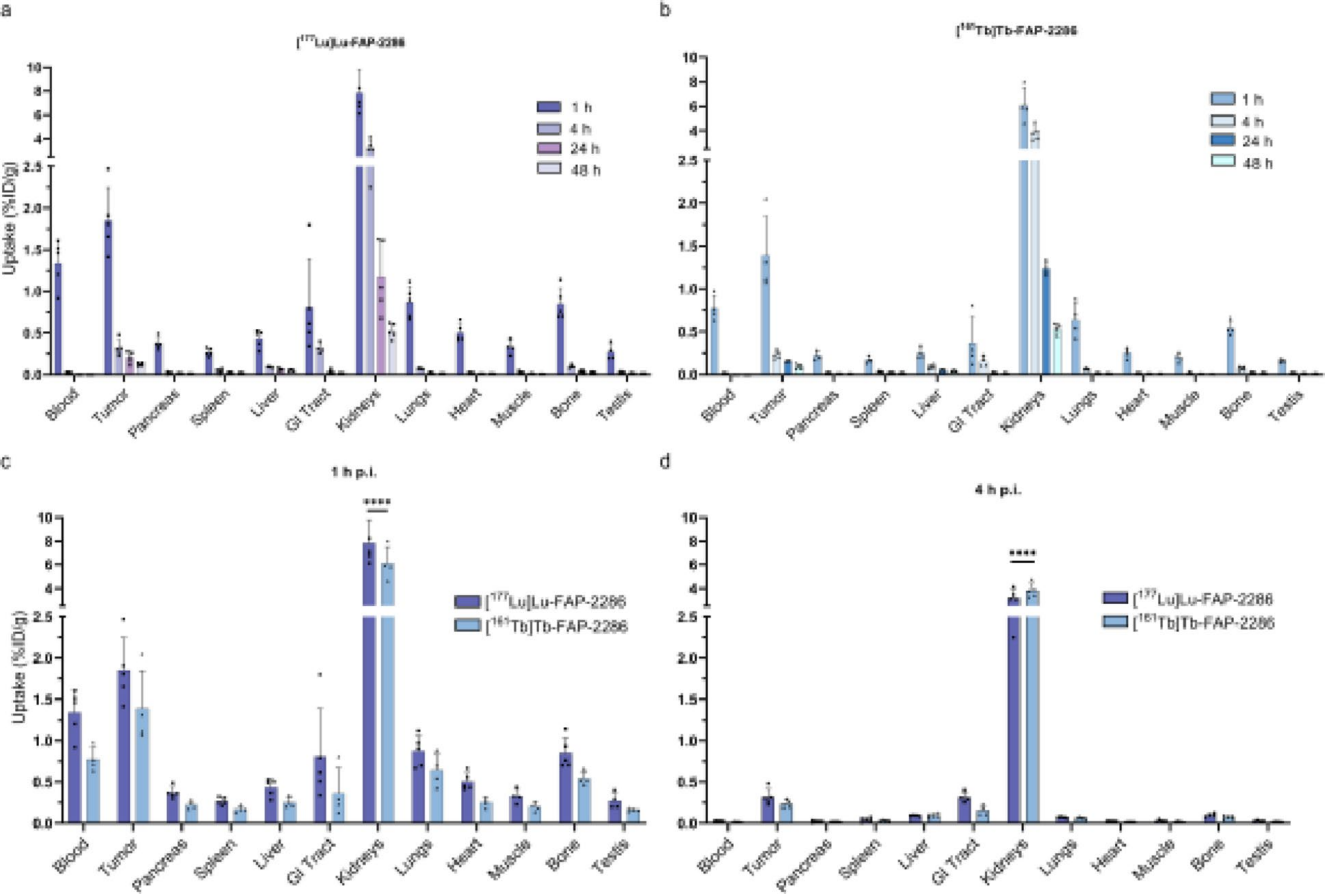
Ex vivo biodistribution of 5 MBq/500 pmol (**a**) [^177^Lu]Lu-FAP-2286 and (**b**) [^161^Tb]Tb-FAP-2286 at 1, 4, 24, and 48 h p.i. (*n* = 4–5). Side-by-side comparison of organ uptake of [^177^Lu]Lu-FAP-2286 and [^161^Tb]Tb-FAP-2286 at (**c**) 1 h and (**d**) 4 h p.i. ^****^ *p* < 0.0001. *%ID/g = percentage injected dose per gram*

Biodistribution of [^177^Lu]Lu-FAP-2286 and [^161^Tb]Tb-FAP-2286 in T110299-xenografted mice was comparable overall; statistical differences were only observed for kidney uptake at 1 and 4 h p.i. A tumor uptake of 1.85 ± 0.39% ID/g for [^177^Lu]Lu-FAP-2286 and 1.39 ± 0.45% ID/g for [^161^Tb]Tb-FAP-2286 at 1 h p.i. was demonstrated. Radiopharmaceutical washout was already observed at 4 h p.i., with only 0.32 ± 0.10% ID/g [^177^Lu]Lu-FAP-2286 and 0.23 ± 0.05% ID/g [^161^Tb]Tb-FAP-2286 remaining at this time point, and further decreasing at 24 h and 48 h p.i. (Fig. 2a-b, Table S4-5). Besides the tumor, the kidneys were the only organ with noticeable radiopharmaceutical uptake, i.e. 7.85 ± 1.96% ID/g at 1 h p.i. and 3.20 ± 0.68% ID/g at 4 h p.i. for [^177^Lu]Lu-FAP-2286, and 6.09 ± 1.41% ID/g at 1 h p.i. and 3.84 ± 0.59% ID/g at 4 h p.i. for [^161^Tb]Tb-FAP-2286 (Fig. 2c-d, Table S4-5). The kidney uptake of [^161^Tb]Tb-FAP-2286 was significantly lower at 1 h p.i. (*p* < 0.001), but significantly higher at 4 h p.i. (*p* < 0.001) compared to that observed with [^177^Lu]Lu-FAP-2286.

Co-injection with UAMC-1110 did not demonstrate a significant decrease in [^177^Lu]Lu-FAP-2286 or [^161^Tb]Tb-FAP-2286 tumor uptake. Only kidney uptake was found to be significantly decreased compared to that of animals injected with only the radiopharmaceutical (Lu-177: 7.85 ± 1.96% (non-blocked) vs. 3.20 ± 0.68% ID/g (blocked), and Tb-161: 3.84 ± 0.59% (non-blocked) vs. 1.84 ± 0.18% ID/g (blocked), *p* < 0.0001) (Fig. S5).

Ex vivo autoradiography on the excised tumors showed results in accordance with the ex vivo biodistribution. However, the ex vivo autoradiography did indicate a significantly lower signal in tumors from the animals co-injected with UAMC-1110 compared to the tumors from mice that only received radiolabeled FAP-2286 (i.e. Lu-177: 9206 ± 2507 (blocked) vs. 23057 ± 3786 DLU/mm^2^ (non-blocked), *p* < 0.01, and Tb-161: 5551 ± 418 (blocked) vs. 15911 ± 3106 DLU/mm^2^ (non-blocked), *p* < 0.01) (Fig. S6). Additionally, an in vitro autoradiography on treatment naïve T110299 tumors, demonstrated that co-incubation with 1000x excess UAMC-1110 significantly blocked binding of [^177^Lu]Lu-FAP-2286 (*p* < 0.05, Fig. S7a, c) and [^161^Tb]Tb-FAP-2286 (*p* < 0.01, Fig. S7b, c).

### Dosimetry

The organ and tumor dosimetry are presented in Table S6. Based on the dosimetry calculations 4 × 40 MBq/500 pmol IV injections were selected for following efficacy studies, corresponding to 4 × 1.5 Gy Lu-177 and 4 × 1.6 Gy Tb-161 (Fig. S8).

### *In vivo efficacy of mono treatment*

**Fig. 3:**
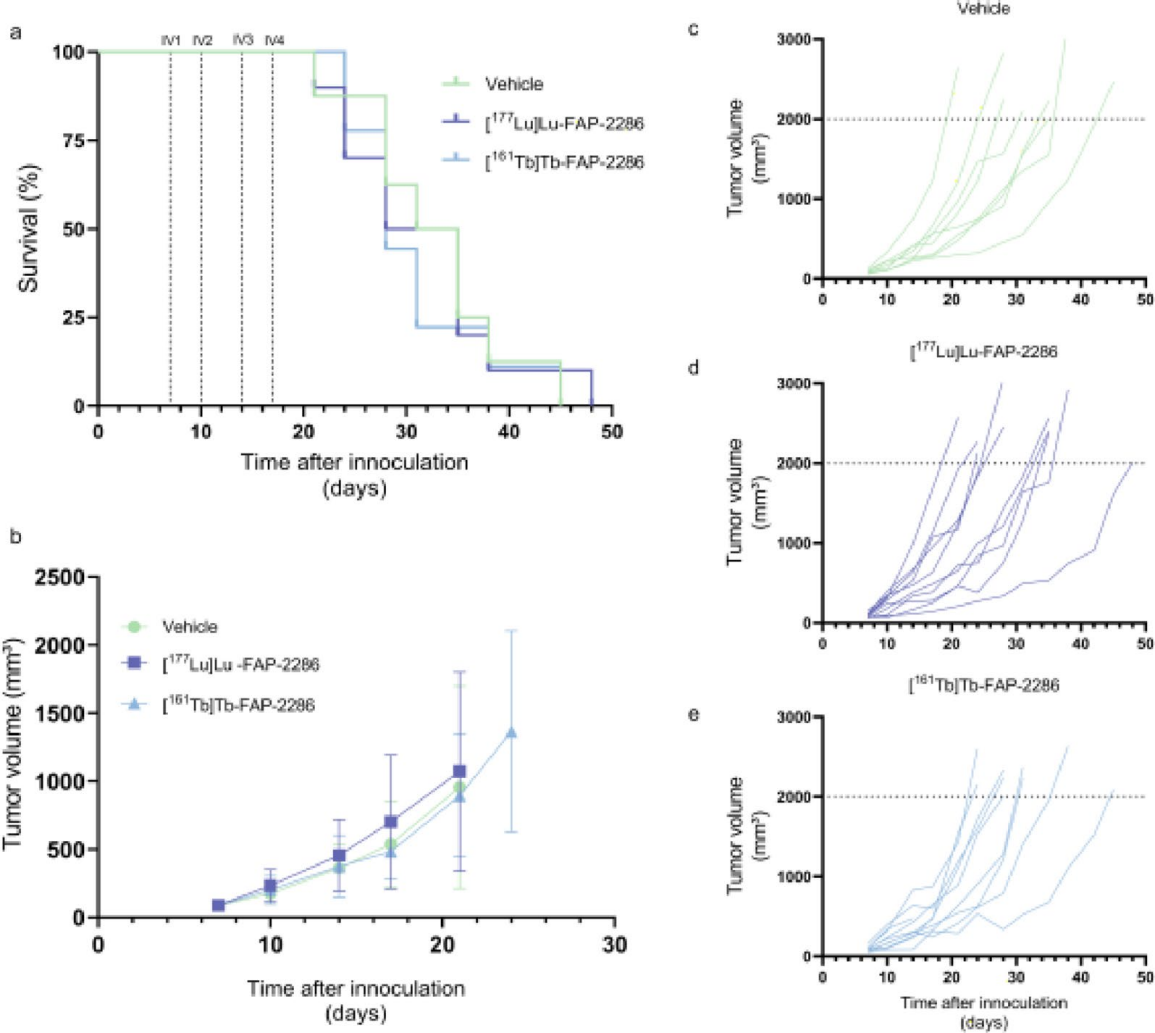
(**a**) Kaplan-Meijer curve of the survival (ns) and (**b**) tumor volume (ns) of mice treated with 4 × 40 MBq/500 pmol [^177^Lu]Lu-/[^161^Tb]Tb-FAP-2286 (*n* = 10/group), or vehicle (*n* = 8). (**c-e**) Tumor volumes of individual animals per treatment group. *ns = non-significant*

**Fig. 4:**
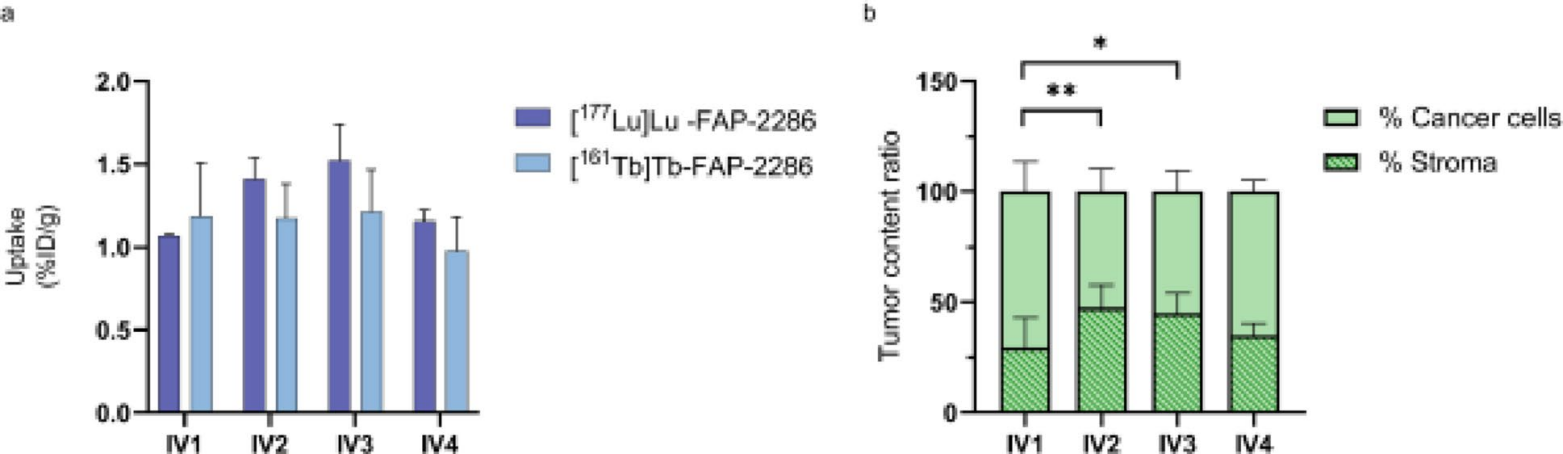
(**a**) Ex vivo tumor uptake of [^177^Lu]Lu-FAP-2286 and [^161^Tb]Tb-FAP-2286 (ns) (*n* = 3/time point) after each intravenous injection (IV) of the radiopharmaceuticals. (**b**) The relative stroma-to-cancer cell ratios after each IV injection (*n* = 8/time point). ^*^p < 0.05, ^**^p < 0.01. *%ID/g = percentage injected dose per gram*,* ns = non-significant*

Treatment with [^177^Lu]Lu-FAP-2286 and [^161^Tb]Tb-FAP-2286 in the T110299-xenografted mice showed no effect on tumor growth (Fig. 3). Radiopharmaceutical uptake in the tumors 1 h p.i. showed an uptake of 1–1.5.5% ID/g for both radiopharmaceuticals, which was in line with the biodistribution studies (Fig. 4a). No significant changes in body weight were observed (Fig. S9a). IHC on tumors harvested 1 h p.i., demonstrated heterogeneity in stroma-to-cancer cell ratios and level of FAP-staining. Accordingly, no differential effect on stroma composition could be distinguished between vehicle, [^177^Lu]Lu-FAP-2286, and [^161^Tb]Tb-FAP-2286 treated mice. Stroma density pooled for all three groups indicated a significant increase in stroma density over time (Fig. 4b, IV to IV2 *p* < 0.01; IV1 to IV3 *p* < 0.05). However, stroma density did not correlate with FAP-expression level, radiopharmaceutical uptake, tumor size, or tumor weight (Fig. S10).

### In vivo efficacy of tandem treatment

**Fig. 5:**
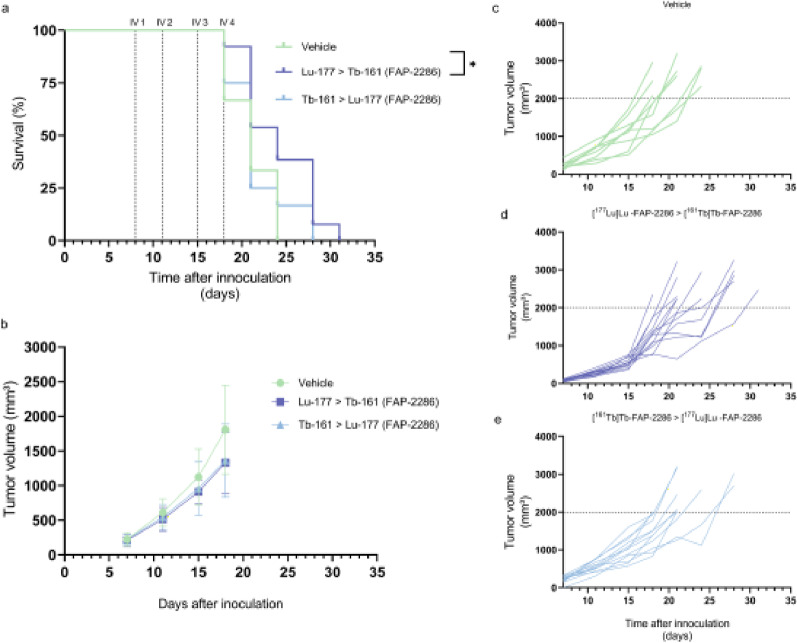
(**a**) Kaplan-Meijer curve of the survival and (**b**) tumor volume of mice treated with 2 × 40 MBq/500 pmol [^177^Lu]Lu-FAP-2286 (*n* = 13) followed by 2×[^161^Tb]Tb-FAP-2286 or vice versa (*n* = 10/group), and vehicle treated mice (*n* = 9). (**c-e**) Tumor volumes of individual animals per treatment group. ^*^ *p* < 0.05

To determine whether tandem therapy has additional value, T110299-xenografted mice were treated with 2 × 40 MBq/500 pmol [^177^Lu]Lu-FAP-2286 followed by 2 × 40 MBq/500 pmol [^161^Tb]Tb-FAP-2286, or vice versa. The Kaplan-Meier survival curve demonstrated a significant longer survival for animals that received 2 × 40 MBq/500 pmol [^177^Lu]Lu-FAP-2286 followed by 2 × 40 MBq/500 pmol [^161^Tb]Tb-FAP-2286 over those that received vehicle (Fig. 5a). Tumor shrinkage was not observed in any of the groups (Fig. 5b-e). In line with the previous treatment study, no significant changes in body weight were observed between the experimental groups (Fig. S9b).

### In vitro autoradiography and accompanying dosimetry

**Fig. 6:**
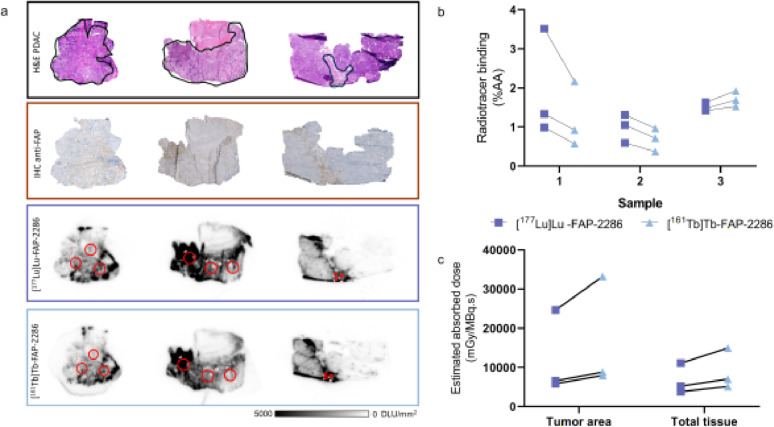
(**a**) Hematoxylin and eosin (H&E) staining of PDAC patient material (tumor area delineated in black) and in vitro autoradiography results after incubation with radiolabeled FAP-2286. (**b**) Quantification of the normalized radiopharmaceutical binding in three red encircled areas for both Lu-177 and Tb-161 (ns). (**c**) Calculated absorbed dose to the tumor area and to the total tissue slice in mGy/MBq.s., *ns = non-significant*

Autoradiography on patient PDAC material demonstrated potent but heterogeneous binding of both [^177^Lu]Lu-FAP-2286 and [^161^Tb]Tb-FAP-2286 (Fig. 6a). Overall, radiopharmaceutical binding was FAP specific. Lower binding was observed for [^161^Tb]Tb-FAP-2286 compared to [^177^Lu]Lu-FAP-2286, but the difference was not significant (Fig. 6b, *p* > 0.05). Dosimetry calculations performed on the autoradiography data resulted in 1.35-fold higher absorbed dose in both the tumor area and to the whole tissue slice with [^161^Tb]Tb-FAP-2286 compared to [^177^Lu]Lu-FAP-2286 (Fig. 6c). The same trends were observed for radiopharmaceutical binding to BC tissue samples (Fig. S11).

## Discussion

Improved efficacy of Tb-161 over Lu-177 for PSMA- and SSTR2-TRT has been reported for the treatment of mCRPC [13, 22, 23] and NETs [14, 24], respectively. Next to enhanced therapeutic efficacy, introducing Tb-161 as substitute or alongside Lu-177 can alleviate the growing supply pressure on Lu-177 [25]. Therefore, we aimed to compare Lu-177 and Tb-161 for FAP-TRT. To our knowledge, this is the first study characterizing and comparing [^177^Lu]Lu-FAP-2286 and [^161^Tb]Tb-FAP-2286.

The radiolabeling of FAP-2286 with both Lu-177 and Tb-161 was successful, and the radiopharmaceuticals remained equally stable in labeling solution and PBS. In contrast, their stability in mouse serum decreased to 70% and 75% after 2 h for Lu-177 and Tb-161, respectively, and further decreased drastically below 15% after 24 h. Nevertheless, due to rapid blood circulation in mice [26], the fast tumor uptake observed, and since > 70% of the radiopharmaceutical remained intact during the first 2 h, the decrease in stable compound is not expected to have had a major effect on radiopharmaceutical uptake in our study.

In vitro cell uptake studies demonstrated that the behavior of [¹⁷⁷Lu]Lu-FAP-2286 and [¹⁶¹Tb]Tb-FAP-2286 was similar regardless of the applied cell model. In line with this, in vitro autoradiography studies showed similar binding of the radiopharmaceuticals to cancer tissues. Thus, the behavior of FAP-2286 does not appear to be altered when radiolabeled with Tb-161 compared to radiolabeling with Lu-177.

In contrast to other studies that used FAP-2286 in vivo in different models, we observed relatively low uptake of the radiopharmaceutical in the T110299 CDX [6, 27, 28]. This is most likely because, unlike models established with cancer cells (over)expressing FAP, the T110299 cells do not express FAP themselves and depend on infiltration of murine FAP (muFAP)-expressing CAFs for FAP-2286 uptake [6]. The T110299 xenografted mouse model has previously been used successfully for evaluation of FAP-targeting interventions [29, 30]; however, model-dependent uptake of FAP-2286 has been reported [27]. Additionally, the injected peptide mass might affect radiopharmaceutical uptake, as illustrated by various recent studies [31, 32], yet there is no consensus on the optimal mass for FAP-2286 or other peptide-based FAP-targeting radiopharmaceuticals. Another explanation for the observed low tumor uptake might be the slightly lower affinity of FAP-2286 for muFAP compared to huFAP (muFAP: 4.7 ± 1.5 nM, huFAP: 1.1 ± 0.5 nM) [6]. FAP-2287, which is similar to FAP-2286 but has higher muFAP affinity [33], was recently developed and should be considered in future studies using models with muFAP-expressing CAFs.

Ex vivo biodistribution studies did not show significantly lower tumor uptake in mice co-injected with UAMC-1110 compared to those injected with [¹⁷⁷Lu]Lu-FAP-2286 or [¹⁶¹Tb]Tb-FAP-2286 alone. Although the injected 35-time excess of UAMC-1110 was insufficient to saturate muFAP and prevent radiolabeled FAP-2286 binding, we believe that the majority of radiolabeled FAP-2286 tumor uptake was FAP-specific. First, performing more sensitive ex vivo autoradiography studies on material from these tumors, we observed significant effective blocking of radiolabeled FAP-2286. Second, in additional in vitro autoradiography studies using untreated T110299 tumor material a 1000× excess of UAMC-1110 did block binding of radiolabeled FAP-2286. Lastly, it has previously been demonstrated in vivo that FAP-2286 binding is FAP-specific, by using a higher excess (e.g. 60-fold) of an unlabeled FAP-targeting compound for blocking [27]. Thus, the UAMC-1110 concentration was likely too low to achieve complete blocking in our in vivo studies, however, evidence suggests that radiolabeled FAP-2286 binding is FAP-specific, and this interpretation is consistent with its ongoing evaluation in a large prospective clinical trial (NCT 04939610). Besides, mice receiving radiolabeled FAP-2286 co-injected with UAMC-1110 showed an unexpected significant decrease in kidney uptake compared to those injected with the radiopharmaceutical alone, even though kidneys do not express FAP. This may be due to altered clearance and reabsorption rates caused by the high concentration of UAMC-1110 present.

Due to the low tumor uptake, dosimetry calculations predicted that a relatively high injected dose of 4 × 40 MBq/500 pmol would be required to achieve up to 50% tumor shrinkage. As this dose could be acquired and a 50% tumor shrinkage would be sufficient to accurately distinguish differences between the two radioisotopes applied, in vivo efficacy studies were performed. However, no survival benefit was observed, apart from a minor growth delay in the tandem therapy arm that first received 2×[¹⁷⁷Lu]Lu-FAP-2286 followed by 2×[¹⁶¹Tb]Tb-FAP-2286. The dosimetry model predicted a marginal advantage for administering Lu-177 followed by Tb-161 compared to the reverse sequence; however, tumor control efficacy remained lower than predicted. This discrepancy is likely due to the dosimetry model not accounting for all biological factors, including heterogeneity of FAP expression, indirect irradiation from CAFs to cancer cells, and differences in radiosensitivity between CAFs and cancer cells. Treated tumors were subjected to IHC to determine whether Lu-177 or Tb-161 had differential effects on stroma-to-cancer cell ratios or FAP expression levels. Unfortunately, due to high heterogeneity in stroma density, it was not possible to distinguish effects of the radiopharmaceuticals. Notably, tumor growth rates differed between the mono and tandem therapy studies, with vehicle-treated mice in the first treatment study reaching the humane endpoint later than those in the later tandem therapy study. This may be due to mice in the tandem therapy group being inoculated at 9 weeks old, compared to 6 weeks old in the mono therapy group. Other studies have shown that the age of CJ54BL/6 mice can impact the immune system development, which in turn affects tumor development and treatment response [34, 35]. Additionally, since cells with a higher division rate are in general more radiosensitive [36], this difference in growth rate may partly explain the differential therapeutic response between the two studies.

So far mixed responses to [¹⁷⁷Lu]Lu-FAP-2286 therapy have been reported in clinical studies [37] and, unfortunately, there is insufficient data on PDAC patients receiving FAP-TRT to directly relate our findings to that of the clinic. The negative outcome of FAP-TRT observed in our study, which used a histopathological representative model, is in contrast with previous promising results in another model [6], which suggests that FAP-TRT efficacy might be model dependent. This finding raises questions about the value of preclinical in vivo studies that use less clinically representative models, as well as about the overall potential of FAP-TRT in PDAC. It should be noted that the heterogeneity in FAP-expression and tumor growth, together with the low radiolabeled FAP-2286 uptake, were limitations specific for our study. To further confirm the value of FAP-TRT in stroma dense PDAC, and elucidate differences between Lu-177 and Tb-161, a model with a higher uptake and corresponding higher response rate is necessary in future studies. CDX models established using a cancer cell line with high FAP expression could be applied, although this would be less representative of patient histopathology and its translational value with regard to radiolabeled FAP-2286 uptake remains unclear. Moreover, a model without CAF infiltration would not allow for discerning effects on CAFs and cancer cells. With regards hereto, a potential effect of the short-range CEs and AEs emitted by Tb-161 on both CAFs and cancer cells can be highly relevant for guiding clinical treatment strategies based on cancer type and stroma density. Next to in vivo models, 2D or 3D co-culture models consisting of cancer cells and CAFs in a density and spatial localization representative for clinical pathology can be of value [38]. Importantly, besides model selection, the impact of targeting CAFs for cancer elimination should be studied further. To accurately do so, improved identification of pro- and anti-tumorigenic CAFs should be established and the effect of treatment on these ‘CAF subtypes’ should be distinguished, as both positive and negative impact of CAF depletion have been reported in literature [39, 40].

In an attempt to gain insights in the clinical uptake of radiolabeled FAP-2286, [^177^Lu]Lu-FAP-2286 and [^161^Tb]Tb-FAP-2286 binding to patient PDAC (and BC) tissue was compared. No significant difference in overall tissue binding was observed between the two radiopharmaceuticals, but high intra- and inter-tumor heterogeneity was noted. Dosimetry calculations showed that the absorbed dose was markedly higher in tumor areas compared to the overall tissue slide, confirming that radionuclide treatment directed at CAFs could effectively irradiate malignant areas. Additionally, dosimetry on cancer tissues predicted that the absorbed dose of [¹⁶¹Tb]Tb-FAP-2286 would consistently be higher, even in areas where its binding was lower than that of [¹⁷⁷Lu]Lu-FAP-2286. However, further studies are needed to determine whether this higher dose, predominantly caused by CEs and AEs, would result in increased efficacy. Future studies using a larger sample set and potentially incorporating more complex 3D dosimetry in subsequent tumor slices will be valuable to better understand the extent to which tumor stroma density and FAP heterogeneity serve as critical determinants of FAP-TRT efficacy.

## Conclusions

Our in vitro data suggests that [^177^Lu]Lu-FAP-2286 and [^161^Tb]Tb-FAP-2286 can be used interchangeably for FAP-TRT. In vivo studies demonstrated that a modest tumor growth delay could be achieved using a tandem therapy strategy, yet the efficacy of FAP-TRT therapy was too limited to determine true differential effects between the two radiopharmaceuticals. Alternative models should be considered for future studies, e.g. in vitro 2D or 3D co-culture models incorporating both CAFs and cancer cells, or in vivo using a mouse PDAC model with similar histopathological clinical resemblance, yet a higher response to FAP-TRT. These studies are necessary to study the effects of Lu-177 and Tb-161 on CAFs and cancer cells when applying FAP-TRT.

## Supplementary Information

Supplementary Material 1

## Data Availability

All available data are described in the manuscript or are available in the Supplementary Information. Additional information can be obtained from the corresponding author upon reasonable request.

## Abbreviations

AE: Auger electron
BC: Breast cancer
CAF: Cancer-associated fibroblast
CE: Conversion electron
CDX: Cell line-derived xenograft
DLU: Digital light units
FAP: Fibroblast activation protein
huFAP: Human FAP
HPLC: High-performance liquid chromatography
IHC: Immunohistochemistry
IC50: Half-Maximal inhibitory concentration
Lu-177: Lutetium-177
LET: Linear energy transfer
mCRPC: Metastatic castration resistant prostate cancer
muFAP: Murine FAP
PBS: Phosphate buffered saline
PDAC: Pancreatic ductal adenocarcinoma
p.i.: Post injection
PSMA: Prostate specific membrane antigen
RCY: Radiochemical yield
RCP: Radiochemical purity
SSTR2: Somatostatin receptor subtype 2
TRT: Targeted radionuclide therapy
Tb-161: Terbium-161
