# Supplementary material for: Head-to-head comparison of [177Lu]Lu-FAP-2286 and [161Tb]Tb-FAP-2286 efficacy in a PDAC mouse model
Source: EJNMMI Res. 2026 Jan 9;16:29. doi: 10.1186/s13550-026-01372-5 (PMC12891299; doi:10.1186/s13550-026-01372-5)
Supplement: Supplementary file 1 — Supplementary Material 1 [file 13550_2026_1372_MOESM1_ESM.docx]

**Supplementary Information:**

**Head-to-head comparison of [^177^Lu]Lu-FAP-2286 and [^161^Tb]Tb-FAP-2286 efficacy in a PDAC model**

Circe D. van der Heide^1^, Carolline M. Ntihabose^1,2^, Mark Konijnenberg^1^, Hanyue Ma^1^, Debra Stuurman^1^, Corrina de Ridder^3^, Yann Seimbille^1^, Michail C. Doukas^4^, Erik de Blois^1^, Simone U. Dalm^1^

^1^Department of Radiology & Nuclear Medicine, Erasmus MC, Rotterdam, The Netherlands

^2^Department of Hospital Pharmacy, Erasmus MC, Rotterdam, The Netherlands.

^3^Department of Experimental Urology, Erasmus MC, Rotterdam, The Netherlands.

^4^Department of Pathology, Erasmus MC, Rotterdam, The Netherlands.

**Supplementary materials & methods**

*Radiochemistry*

The molar activity for in vitro studies and in vivo biodistribution studies ranged from 20-80 MBq/nmol and all labeling solutions were prepared in 1.5 mL microtubes (Sarsted, Etten-Leur, The Netherlands). The radiolabeling solutions for in vitro studies contained water with Kolliphor® HS 15 (2 mg/mL) (Sigma Aldrich, Saint-Louis MS, USA) to prevent stickiness, sodium acetate (1 µL, 2.5 M), and quenchers (ascorbic acid/gentisic acid 10 µL, 50 mM and L-methionine 10 µL, 50 mM) to minimize radiolysis. These mixtures were incubated at 90 °C for 20 min and then left to cool down for 5 min. Next, 5 µl of diethylenetriaminepentaacetic acid (DTPA; 3 mg/mL) was added to chelate the unbound radionuclide, and 3.5 µL 5% Tween® 20 (Sigma Aldrich, Saint-Louis MS, USA) was added to improve solubility of the radiopharmaceuticals.

For in vivo therapy studies, the labelling mixture consisted of FAP-2286 with either Lu-177 or Tb-161 at a molar activity of 80 MBq/nmol. The mixture was prepared with water containing Koliphor® HS 15 (2 mg/mL), sodium acetate (2 µL, 2.5 M), and quenchers (ascorbic acid/gentisic acid 20 µL, 50 mM and L-methionine 20 µL, 50 mM). The stability of the labeling mixture was confirmed using high-performance liquid chromatography (HPLC) (Alliance 2695 XE, Waters™, Etten Leur, The Netherlands). The activities were measured using VIK-202 and VDC 404 dose calibrators (Comecer, Castel Bolognese, Italy). Prior to every assay radiochemical yield was determined by instant-thin-layer chromatography (iTLC) prior to use. Labeling of [^177^Lu]Lu-FAP-2286 and [^161^Tb]Tb-FAP-2286 gave a radiochemical yield (RCY) of >98% and a radiochemical purity of (RCP) of >97%.

*Quality control of [^177^Lu]Lu-FAP-2286 and [^161^Tb]Tb-FAP-2286*

The RCY was determined by instant-thin-layer chromatography (iTLC) on silica gel coated glass fiber iTLC-SG sheets (Agilent, Folsom CA, USA). The plates were cut into 0.5 x 10 cm strips and dried for 20 min at 200 °C prior to usage. Elution was performed using a sodium citrate solution (0.1 M, pH 5). The RCP was determined by HPLC using an Alliance 2695 XE HPLC (Waters™, Etten-Leur, The Netherlands). It included a W2998 PDA detector, a 1 inch NaI(TI) Scionic crystal (Bunnik, The Netherlands) connected to a Bpad single channel analyzer radiodetector and signal amplifier (Brightspec, Zellik, Belgium). The HPLC system was operated using Empower 3 software (Waters™). Samples were injected onto an analytical C18 Symmetry® column (Waters™) (250.0 x 4.6 mm, 5 µm) eluted with a gradient of methanol (containing 0.1% trifluoroacetic acid) with a flowrate of 1 mL/min for 25 min. Region of interest and retention time were determined by iTLC and HPLC, respectively, for [^177^Lu]Lu-FAP-2286 and [^161^Tb]Tb-FAP-2286, while [^177^Lu]Lu-DTPA and [^161^Tb]Tb-DTPA were used as control.

*Stability studies*

Stability was determined using an Acquity Arch system equipped with a diode array detector, a radioactivity detector, a Canberra Osprey multichannel analyzer, Empower 3 software and an analytical C18 Symmetry® column (Waters™) (250.0 x 4.6 mm, 5 µm). The solution was eluted with a gradient of methanol (0-100% in H_2_O containing 0.1% trifluoroacetic acid) with a flowrate of 1 mL/min for 25 min. Region of interest and retention time were determined by iTLC and HPLC, respectively, for [^177^Lu]Lu-FAP-2286 and [^161^Tb]Tb-FAP-2286, while [^177^Lu]Lu-DTPA and [^161^Tb]Tb-DTPA were used as control. During the validation of the method, the retention time of the radiopharmaceuticals (13.5-13.9 min), radiolyzed radiopharmaceutical (13.00-13.50 min), and unbound/[^177^Lu]Lu-DTPA/[^161^Tb]Tb-DTPA (2.00-3.00 min) were determined. After 2 and 24 h of incubation in PBS, the samples were injected onto the radio-UPLC. For the radiopharmaceuticals incubated in mouse serum, first 30 µL of the mixture was transferred to a new Eppendorf tube, and the proteins were precipitated by adding 30 µL of acetonitrile. Following the tubes were vortexed vigorously and centrifuged (5000x g) at 4 °C for 20 min and subsequently injected onto the radio-UPLC.

*Affinity*

To determine whether radiolabeling with Lu-177 and Tb-161 affected the affinity of FAP-2286, a competition binding assay was performed on HT1080-huFAP cells. This resulted in a similar competition binding curve and IC_50_ for both compounds, which was 2.9 ± 7.2 nM for [^177^Lu]Lu-FAP-2286 and 1.5 ± 3.2 nM for [^161^Tb]Tb-FAP-2286.

*Animal studies*

For biodistribution studies, C57/BL6 male mice (7 weeks old) were inoculated with T110299 cells, and tumors were allowed to grow for 11 days, resulting in an average tumor volume of 431 ± 173 mm^3^ and 315 ± 159 mm^3^ for the mice receiving Lu-177 and Tb-161, respectively (Fig. S1). For monotherapy efficacy studies, mice (6 weeks old) were inoculated with T110299 cells, and tumors were grown for 7 days to reach an average tumor size of 89 ± 38 mm^3^. IV injections were administered on day 7, 10, 14, and 17 after T110299 cell inoculation. In the tandem therapy study mice were inoculated at 9 weeks old. Tumors were left to grow for 7 days, reaching an average tumor size of 219 ± 83 mm^3^, and the four IV injections were administered on day 8, 11, 15, and 18 after T110299 cell inoculation.

Animals were monitored multiple days per week, and the tumor size was measured twice a week using a caliper. Tumor volume was calculated by using the following formula: π/6(tumor length ×tumor width)^1.5^. Animals were euthanized when tumor size was ≥2000 mm^3^ or when other preset humane endpoint criteria were met. For all animals (half of the) tumors and kidneys were collected and directly fixated in 10% buffered formalin after euthanasia or snap frozen in liquid nitrogen. During one study, one mouse died during handling at day 14, and was therefore excluded from the analyses.

*Immunohistochemistry*

Formalin-fixed paraffin embedded (FFPE) tissue slices of the T110299 xenografts were stained with hematoxylin and eosin (H&E) and were subjected to a immunohistochemistry (IHC) DAB staining with anti-FAP polyclonal antibody EPR20021 (Abcam, Cambridge, UK) in 1/50 dilution as previously described [1].

*Supplemental Table S1.* Groups and numbers of C57BL/6 male mice used in biodistribution studies.

| Experiment | Groups | | Number of animals | Age at inoculation of T110299 cells |
| --- | --- | --- | --- | --- |
| Biodistribution | 1 h | [^177^Lu]Lu-FAP-2286 | 5 | 6 weeks |
|  |  | [^161^Tb]Tb-FAP-2286 | 5 | 6 weeks |
|  | 4 h | [^177^Lu]Lu-FAP-2286 | 5 | 6 weeks |
|  |  | [^161^Tb]Tb-FAP-2286 | 5 | 6 weeks |
|  | 24 h | [^177^Lu]Lu-FAP-2286 | 5 | 6 weeks |
|  |  | [^161^Tb]Tb-FAP-2286 | 5 | 6 weeks |
|  | 48 h | [^177^Lu]Lu-FAP-2286 | 5 | 6 weeks |
|  |  | [^161^Tb]Tb-FAP-2286 | 5 | 6 weeks |
|  | 4 h + block | [^177^Lu]Lu-FAP-2286 | 4 | 6 weeks |
|  |  | [^161^Tb]Tb-FAP-2286 | 4 | 6 weeks |

*Supplemental Table S2.* Groups and numbers of C57BL/6 male mice used in therapy studies.

| Experiment | Groups | Number of animals | Age at inoculation of T110299 cells |
| --- | --- | --- | --- |
| Therapy efficacy (single) | [^177^Lu]Lu-FAP-2286 | 10 | 6 weeks |
|  | [^161^Tb]Tb-FAP-2286 | 10 | 6 weeks |
|  | Vehicle | 8 | 6 weeks |
| Therapy effect on immunohistochemistry (single) | [^177^Lu]Lu-FAP-2286 | 12 | 6 weeks |
|  | [^161^Tb]Tb-FAP-2286 | 12 | 6 weeks |
|  | Vehicle | 8 | 6 weeks |
| Therapy efficacy tandem | [^177^Lu]Lu-FAP-2286 > [^161^Tb]Tb-FAP-2286 | 13 | 9 weeks |
|  | [^161^Tb]Tb-FAP-2286  > [^177^Lu]Lu-FAP-2286 | 13 | 9 weeks |
|  | Vehicle | 9 | 9 weeks |

*Statistics*

Prior to statistical analyses, data was always tested for normality, and outliers were first removed using a Grubbs’ test (α = 0.05). The stability was compared per solution using an unpaired multiple t test. Binding competition curves were determined with a non-linear regression Log(inhibitor) vs. response analysis with variable slope, which was used to determine the IC_50_ values. For in vitro and ex vivo radiopharmaceutical studies, a 2-way ANOVA was performed to test for differences in uptake and localization of the radiopharmaceutical. For the ex vivo biodistribution data, differences in organ uptake between the two radiopharmaceuticals were tested for significance with multiple unpaired t-tests. Survival benefit was determined using a Kaplan-Meijer curve and statistical test. Radiotracer binding on the autoradiography was analyzed for the pooled samples, thus not per tissue, and tested with a Paired t test when data was normally distributed (BC samples) or a Wilcoxon test when non-normal distribution was observed (i.e. PDAC samples).

**Supplementary data**

*Quality control [^177^Lu]Lu-FAP-2286 and [^161^Tb]Tb-FAP-2286*

[^177^Lu]Lu-DTPA and [^161^Tb]Tb-DTPA peaks showed a retention time of 2-3 min, while [^177^Lu]Lu-FAP-2286 and [^161^Tb]Tb-FAP-2286 peaked at ~14 min. To assess radiolysis, the radiolabeling was performed without the addition of quenchers. Quality control over time (24 h) showed the presence of peaks at ~13 min, indicating the degradation of the radiopharmaceuticals by radiolysis. These degradation products were clearly differentiated from the intact radiopharmaceuticals.

b

a


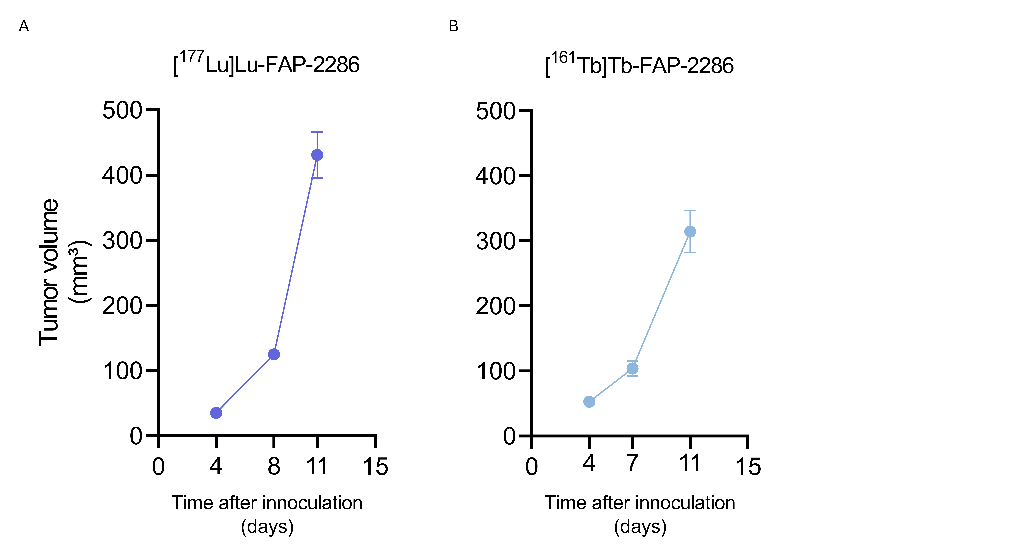


*Supplemental Fig. S1.* Tumor growth of C57/BL6 mice inoculated with T110299 cells that received (a) [^177^Lu]Lu-FAP-2286 and (b) [^161^Tb]Tb-FAP-2286 for biodistribution studies.


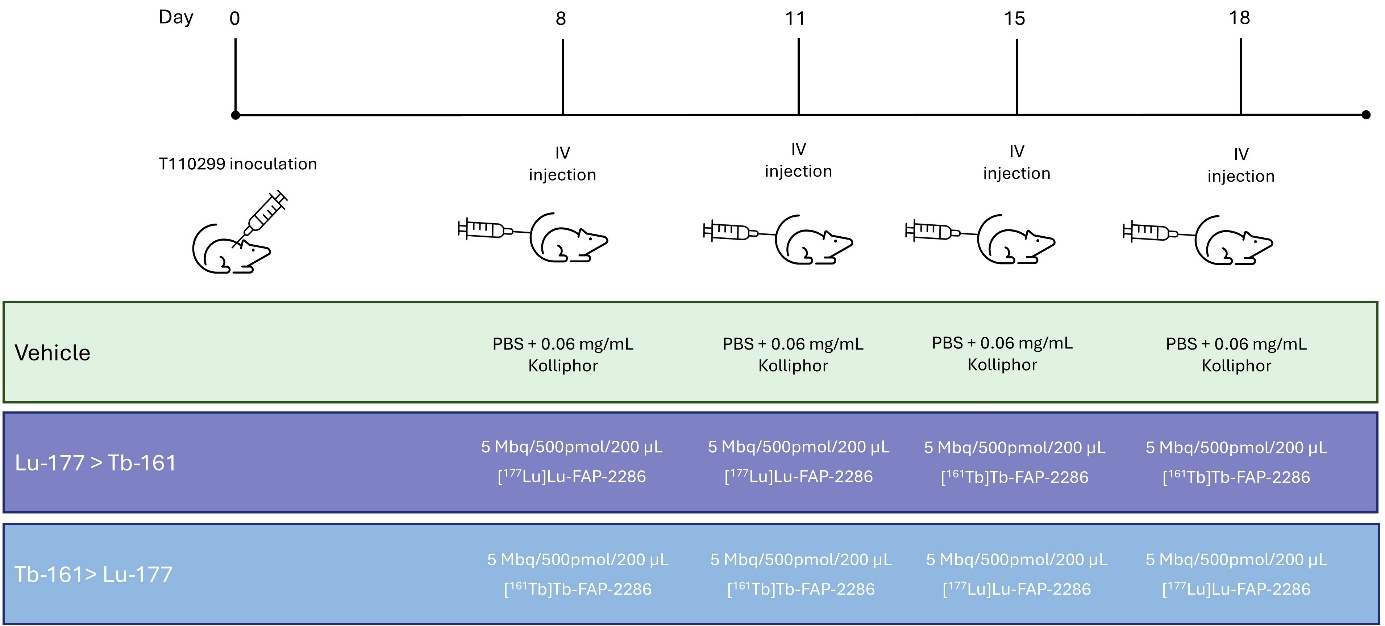


*Supplemental Fig. S2.* Timeline of C57/BL6 mice inoculated with T110299 cells that received tandem therapy with vehicle, sequential [^177^Lu]Lu-FAP-2286 and [^161^Tb]Tb-FAP-2286, or vice versa.


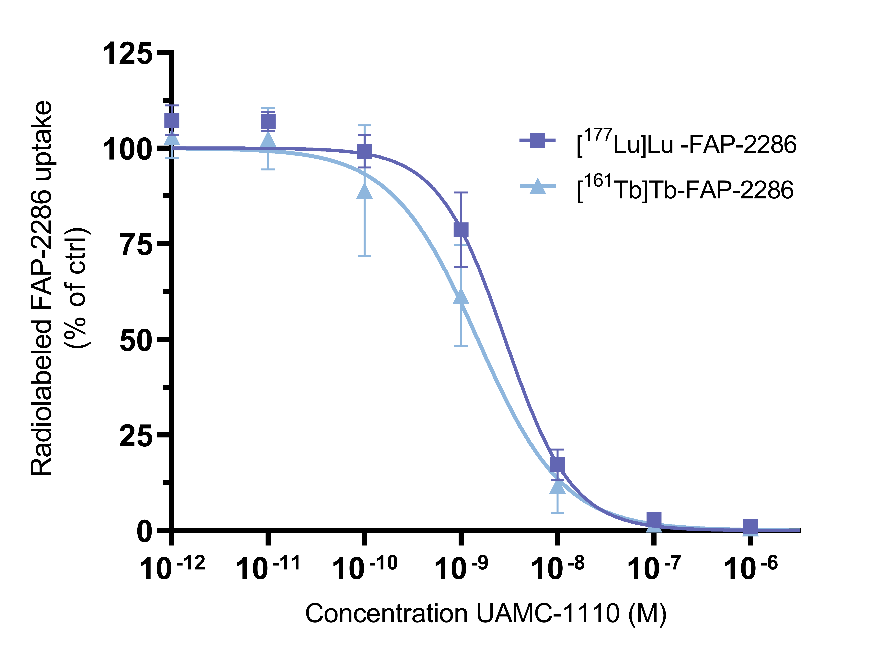


*Supplemental Fig. S3* Competition binding assay on HT1080-huFAP cells using 1 nM [^177^Lu]Lu-FAP-2286 (n=3) or [^161^Tb]Tb-FAP-2286 (n=4), simultaneous with increasing concentrations of UAMC-1110. Data are normalized to the uptake without UAMC-1110, and expressed as mean ± SD.

*Supplemental Table S3.* Stability of [^161^Tb]Tb-FAP-2286 and [^177^Lu]Lu-FAP-2286 (50 MBq/nmol) in labeling solution, PBS and mouse serum over time (n=3).

| Compound | Labeling solution | | | PBS | | Mouse serum | |
| --- | --- | --- | --- | --- | --- | --- | --- |
|  | **0 h** | **2 h** | **24 h** | **2 h** | **24 h** | **2 h** | **24 h** |
| [^177^Lu]Lu-FAP-2286 | 97.7 ±1.3% | 97.6 ±0.6% | 93.4 ±2.1% | 98.1 ±0.7% | 95.7 ±0.5% | 70.0 ±0.5% | 13.0 ±0.5% |
| [^161^Tb]Tb-FAP-2286 | 97.1 ±0.4% | 95.2 ±2.8% | 93.4 ±2.6% | 97.3 ±0.6% | 94.6 ±0.2% | 75.8 ±1.0% | 12.2 ±0.5% |
| *p value* | *0.703* | *0.144* | *>0.999* | *0.104* | *0.036* | *0.000005* | *0.177* |

**
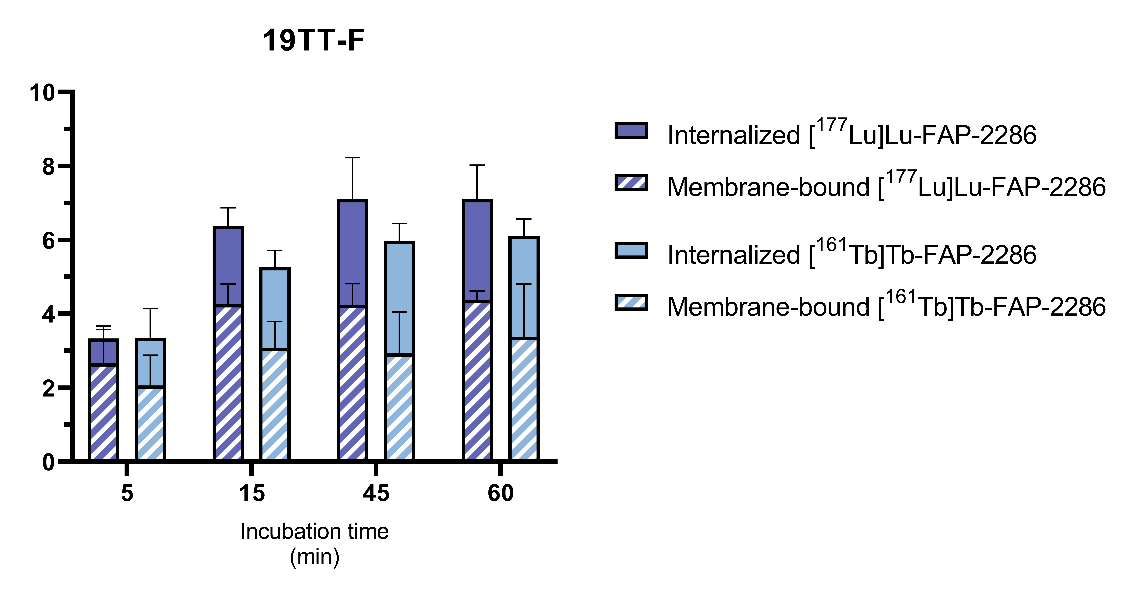
**

*Supplemental Fig. S4.* Uptake of 1 nM radiolabeled FAP-2286 by 19TT-F breast cancer fibroblasts (n=3).

**
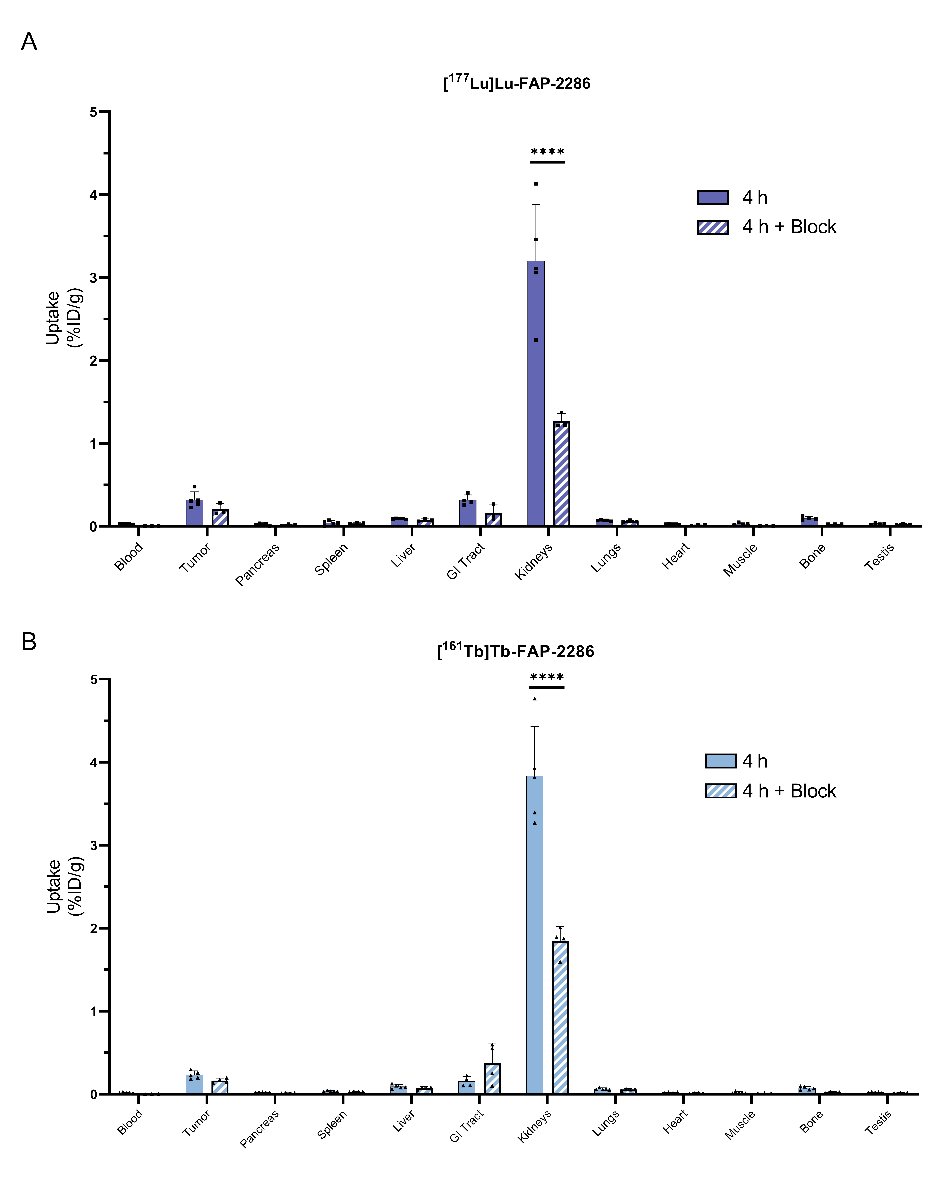
**

b

a

*Supplemental Fig. S5.* Ex vivo biodistribution of 1 nM (a) [^177^Lu]Lu-FAP-2286 and (b) [^161^Tb]Tb-FAP-2286 4 h p.i. without or with co-injection of UAMC-1110 (Block). ^****^ *p* < 0.0001





*Supplemental Fig. S6.* Ex vivo autoradiography of T110299 xenografts collected after biodistribution studies, demonstrating the quantification of bound (a) [^177^Lu]Lu-FAP-2286 and (c) [^161^Tb]Tb-FAP-2286 at 1, 4, 24, and 48 h p.i. A comparison of bound (b) [^177^Lu]Lu-FAP-2286 and (d) [^161^Tb]Tb-FAP-2286 at 4 h without and with block (UAMC-1110). Binding was significantly lower in the presence of UAMC-1110, determined by an unpaired t-test. ^**^ *p* < 0.01.

*Supplemental Table S4.* Biodistribution of [^177^Lu]Lu-FAP-2286 in T110299 xenografted mice.

|  | 1 h | | | 4 h | | | 4 h + Block | | | 24 h | | | 48 h | | |
| --- | --- | --- | --- | --- | --- | --- | --- | --- | --- | --- | --- | --- | --- | --- | --- |
|  | **Mean** | **SD** | **N** | **Mean** | **SD** | **N** | **Mean** | **SD** | **N** | **Mean** | **SD** | **N** | **Mean** | **SD** | **N** |
| Blood | 1.34 | 0.28 | 5 | 0.03 | 0.00 | 5 | 0.01 | 0.00 | 3 | 0.00 | 0.00 | 5 | 0.00 | 0.00 | 5 |
| Tumor | 1.85 | 0.39 | 5 | 0.32 | 0.10 | 5 | 0.21 | 0.07 | 3 | 0.20 | 0.07 | 5 | 0.12 | 0.02 | 5 |
| Pancreas | 0.37 | 0.07 | 5 | 0.02 | 0.01 | 5 | 0.02 | 0.01 | 3 | 0.01 | 0.00 | 5 | 0.01 | 0.00 | 5 |
| Spleen | 0.27 | 0.05 | 5 | 0.04 | 0.03 | 5 | 0.03 | 0.01 | 3 | 0.03 | 0.00 | 5 | 0.02 | 0.00 | 5 |
| Liver | 0.43 | 0.11 | 5 | 0.09 | 0.01 | 5 | 0.07 | 0.01 | 3 | 0.06 | 0.02 | 5 | 0.05 | 0.01 | 5 |
| GI tract | 0.81 | 0.58 | 5 | 0.32 | 0.06 | 4 | 0.16 | 0.09 | 3 | 0.04 | 0.02 | 5 | 0.02 | 0.00 | 5 |
| Kidneys | 7.85 | 1.96 | 5 | 3.20 | 0.68 | 5 | 1.27 | 0.09 | 3 | 1.17 | 0.43 | 5 | 0.53 | 0.09 | 5 |
| Lungs | 0.87 | 0.19 | 5 | 0.07 | 0.01 | 5 | 0.06 | 0.01 | 3 | 0.02 | 0.01 | 5 | 0.01 | 0.00 | 4 |
| Heart | 0.50 | 0.10 | 5 | 0.03 | 0.00 | 5 | 0.01 | 0.00 | 3 | 0.01 | 0.00 | 4 | 0.01 | 0.00 | 5 |
| Muscle | 0.33 | 0.08 | 4 | 0.03 | 0.01 | 4 | 0.01 | 0.00 | 3 | 0.01 | 0.00 | 5 | 0.01 | 0.00 | 5 |
| Bone | 0.85 | 0.19 | 5 | 0.10 | 0.02 | 4 | 0.03 | 0.00 | 3 | 0.04 | 0.01 | 5 | 0.03 | 0.01 | 5 |
| Testis | 0.27 | 0.09 | 4 | 0.03 | 0.01 | 4 | 0.02 | 0.01 | 3 | 0.02 | 0.00 | 5 | 0.01 | 0.00 | 5 |

*Supplemental Table S5.* Biodistribution of [^161^Tb]Tb-FAP-2286 in T110299 xenografted mice.

|  | 1 h | | | 4 h | | | 4 h + Block | | | 24 h | | | 48 h | | |
| --- | --- | --- | --- | --- | --- | --- | --- | --- | --- | --- | --- | --- | --- | --- | --- |
|  | **Mean** | **SD** | **N** | **Mean** | **SD** | **N** | **Mean** | **SD** | **N** | **Mean** | **SD** | **N** | **Mean** | **SD** | **N** |
| Blood | 0.77 | 0.15 | 4 | 0.02 | 0.01 | 5 | 0.00 | 0.00 | 3 | 0.00 | 0.00 | 4 | 0.00 | 0.00 | 5 |
| Tumor | 1.39 | 0.45 | 4 | 0.23 | 0.05 | 5 | 0.17 | 0.03 | 4 | 0.16 | 0.00 | 4 | 0.10 | 0.03 | 5 |
| Pancreas | 0.22 | 0.05 | 4 | 0.03 | 0.00 | 5 | 0.02 | 0.00 | 4 | 0.01 | 0.00 | 5 | 0.01 | 0.00 | 5 |
| Spleen | 0.17 | 0.04 | 4 | 0.04 | 0.01 | 5 | 0.03 | 0.00 | 4 | 0.03 | 0.00 | 5 | 0.02 | 0.00 | 5 |
| Liver | 0.25 | 0.06 | 4 | 0.09 | 0.02 | 5 | 0.07 | 0.02 | 4 | 0.05 | 0.00 | 5 | 0.05 | 0.01 | 5 |
| GI tract | 0.36 | 0.30 | 4 | 0.16 | 0.06 | 4 | 0.38 | 0.24 | 4 | 0.03 | 0.00 | 5 | 0.02 | 0.00 | 5 |
| Kidneys | 6.09 | 1.41 | 4 | 3.84 | 0.59 | 5 | 1.84 | 0.18 | 4 | 1.24 | 0.07 | 5 | 0.54 | 0.06 | 5 |
| Lungs | 0.64 | 0.20 | 4 | 0.06 | 0.01 | 5 | 0.06 | 0.01 | 4 | 0.02 | 0.00 | 5 | 0.01 | 0.00 | 5 |
| Heart | 0.25 | 0.06 | 4 | 0.02 | 0.00 | 4 | 0.01 | 0.00 | 4 | 0.01 | 0.00 | 5 | 0.01 | 0.00 | 5 |
| Muscle | 0.20 | 0.05 | 4 | 0.02 | 0.01 | 5 | 0.01 | 0.00 | 3 | 0.01 | 0.00 | 5 | 0.01 | 0.00 | 5 |
| Bone | 0.54 | 0.08 | 4 | 0.07 | 0.02 | 5 | 0.03 | 0.01 | 4 | 0.03 | 0.00 | 5 | 0.02 | 0.00 | 5 |
| Testis | 0.16 | 0.02 | 4 | 0.02 | 0.00 | 5 | 0.02 | 0.00 | 3 | 0.01 | 0.00 | 5 | 0.01 | 0.00 | 5 |

*Supplemental Table S6.* Predicted dose activity in murine organs expressed in mGy/MBq based on biodistribution studies.

|  | [^161^Tb]Tb-FAP-2286 | [^177^Lu]Lu-FAP-2286 |
| --- | --- | --- |
| Tumor | 40.64 | 37.56 |
| Brain | 2.91 | 2.45 |
| Large intestine | 14.47 | 11.52 |
| Small intestine | 8.17 | 6.92 |
| Stomach wall | 9.94 | 7.44 |
| Heart | 7.06 | 5.60 |
| Kidneys | 260.97 | 183.66 |
| Liver | 17.61 | 14.09 |
| Lungs | 9.90 | 7.94 |
| Pancreas | 8.78 | 6.97 |
| Skeleton | 18.76 | 17.61 |
| Spleen | 10.61 | 7.53 |
| Thyroid | 9.85 | 7.67 |
| Bladder | 2.89 | 2.37 |
| Total body | 8.97 | 7.38 |

**
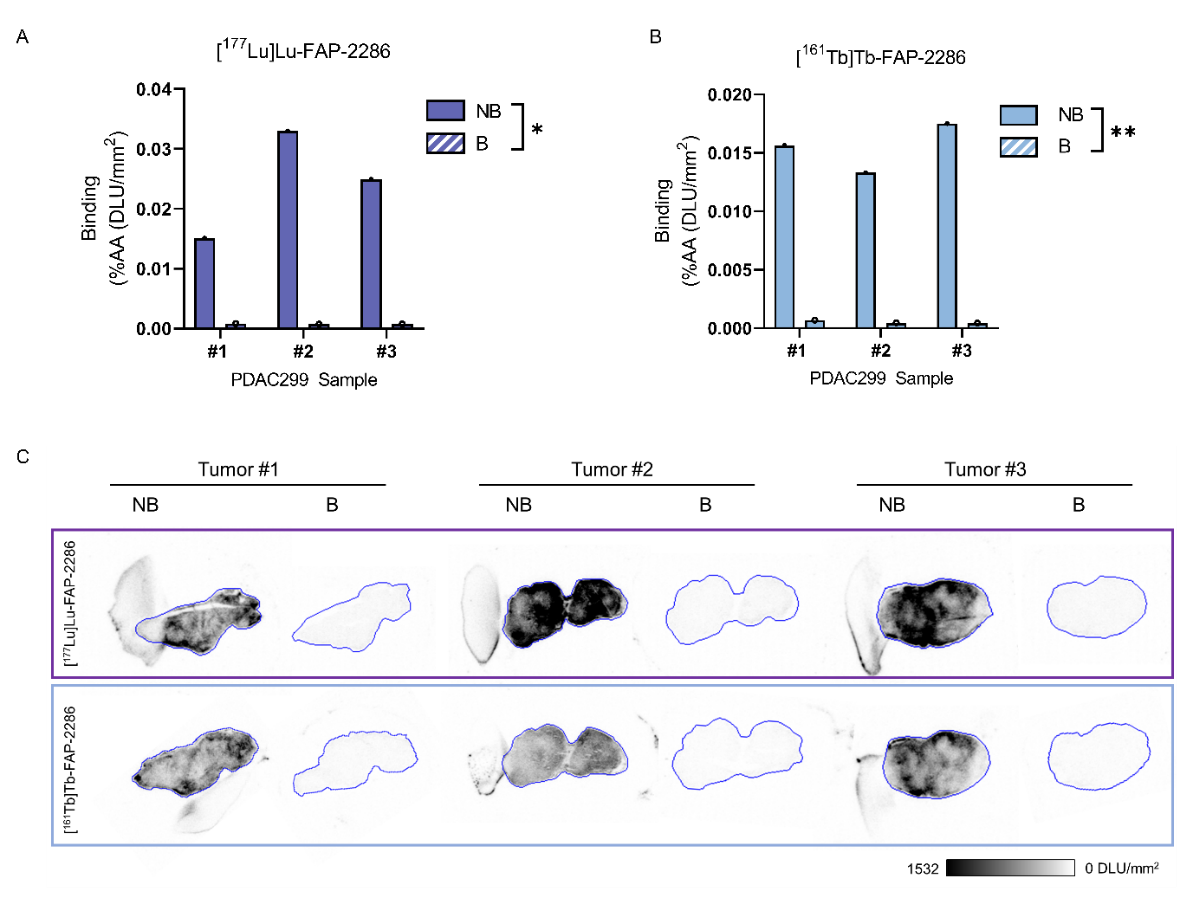
**

c

a

b

*Supplemental Fig. S7.* In vitro autoradiography on treatment naïve T110299 material incubated with (a) 1 nM [^177^Lu]Lu-FAP-2286 or (b) [^161^Tb]Tb-FAP-2286 in the absence (non-blocked (NB)) or presence (blocked (B)) of 1 mM UAMC-1110 quantified as % added activity and (c) a visual of the autoradiography. ^*^ *p* < 0.05. ^**^ *p* < 0.01

a


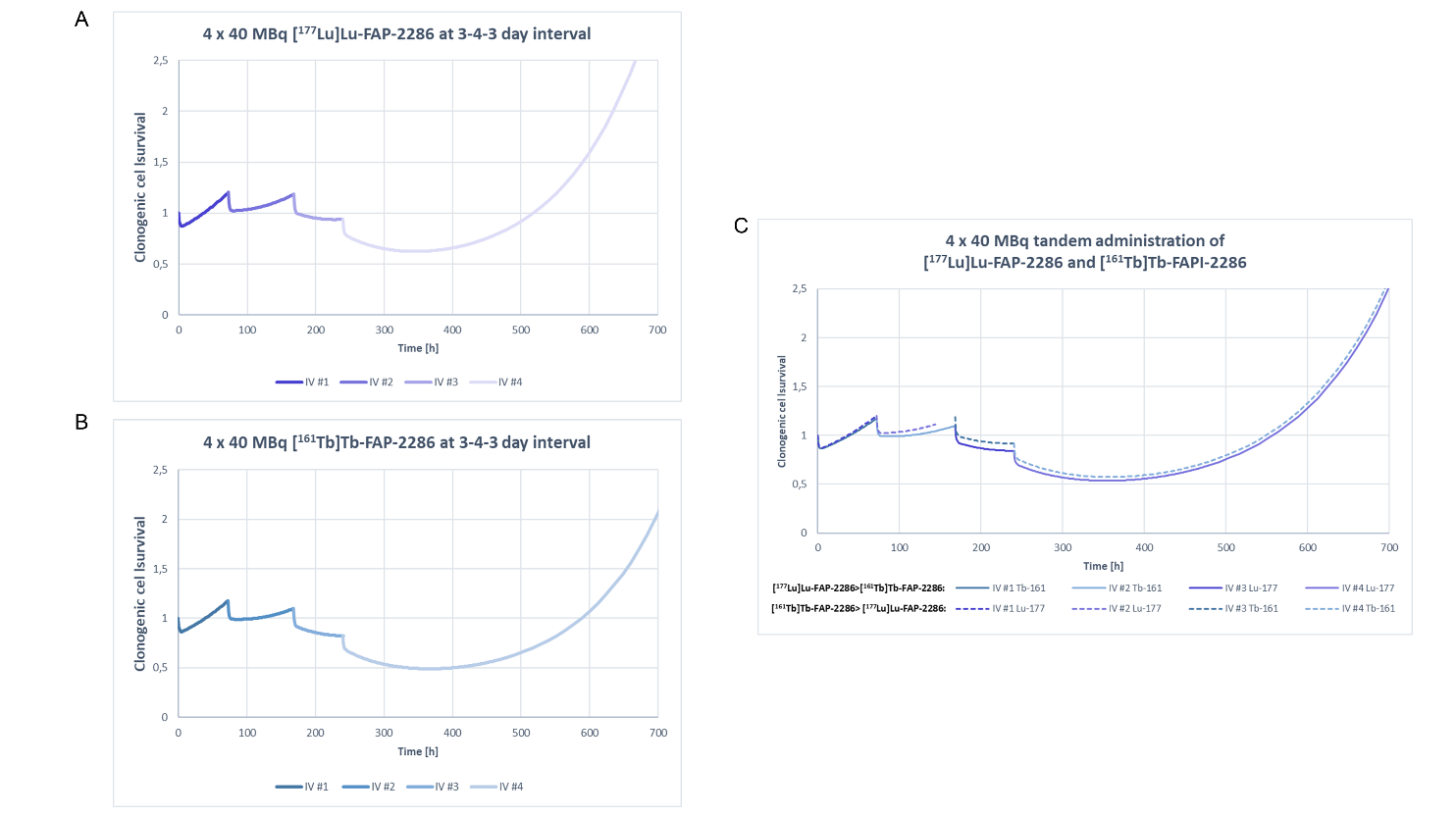


c

b

*Supplemental Fig. S8.* Tumor control probability calculated for four consecutive IV injections of (a) [^177^Lu]Lu-FAP-2286, (b) [^161^Tb]Tb-FAP-2286 and (c) tandem therapy of both radiopharmaceuticals, based on a doubling time (TD) of 3 days, and a cell radiosensitivity (𝛼) of 1.0 /Gy.


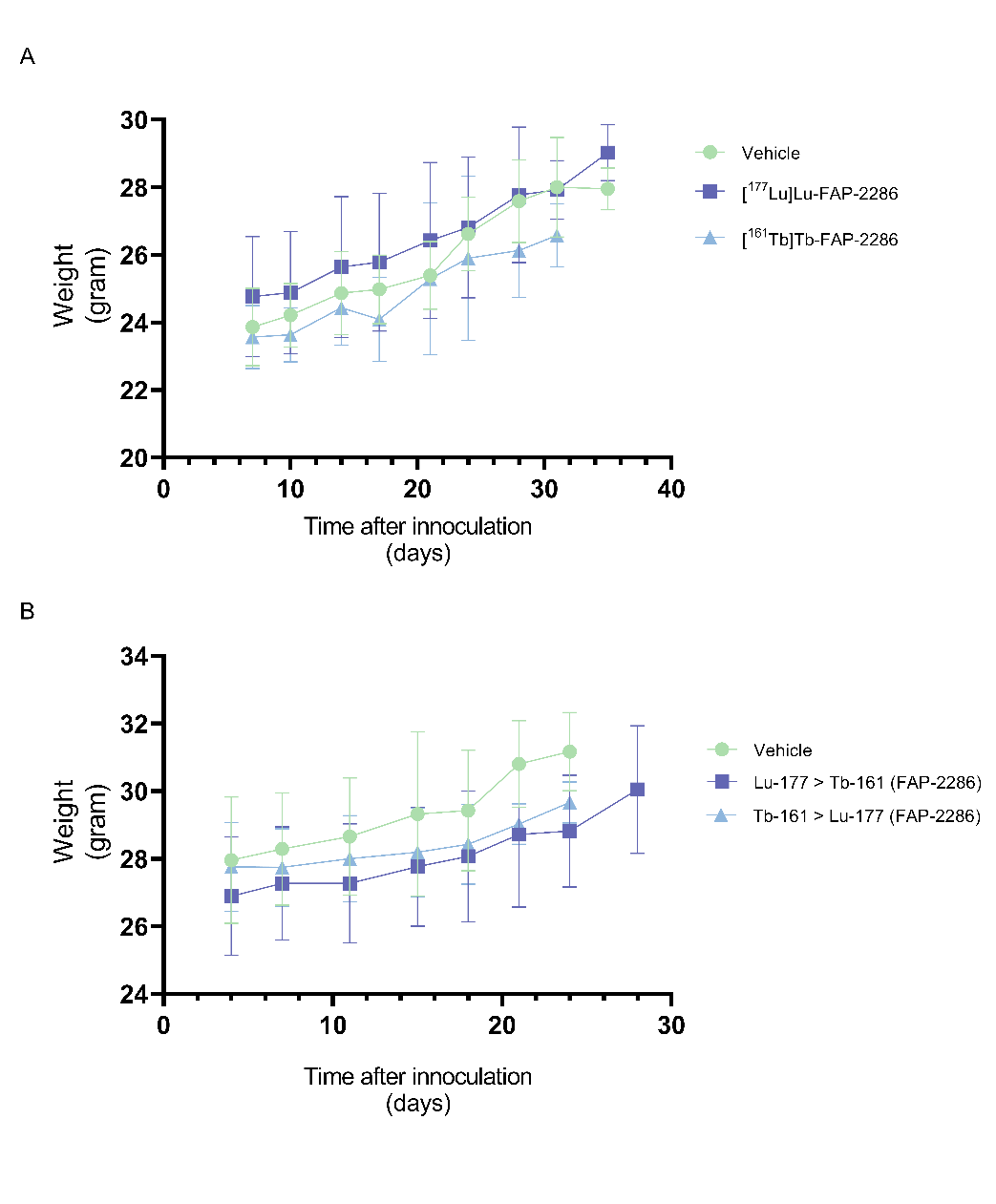


a

b

*Supplemental Fig. S9.* (a) Average weight of mice receiving 4×40 MBq/500 pmol [^177^Lu]Lu-FAP-2286, 4×40 MBq/500 pmol [^161^Tb]Tb-FAP-2286, or vehicle injections. (b) Average weight of mice receiving tandem therapy with 2×40 MBq/500 pmol [^177^Lu]Lu-FAP-2286 followed by 2×40 MBq/500 pmol [^161^Tb]Tb-FAP-2286, vice versa, or vehicle injections.


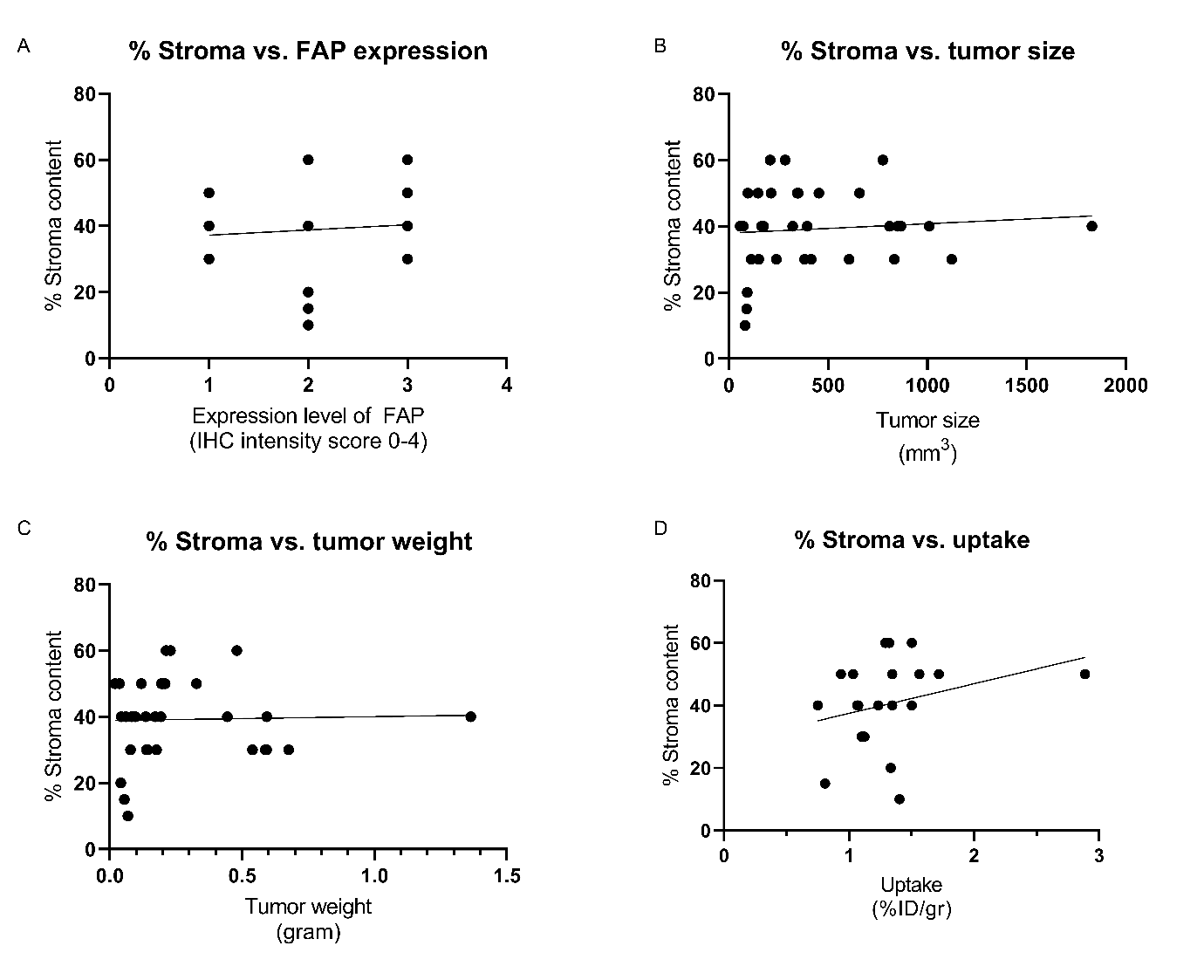


a

c

d

b

*Supplemental Fig. S10.* Correlation of stroma density of the tumors collected after IV1-IV4 with (a) the level of FAP expression (scored between 0-4 based on level of intensity), (b) the tumor size as measured by caliper, (c) the tumor weight ex vivo, and (d) radiopharmaceutical uptake.


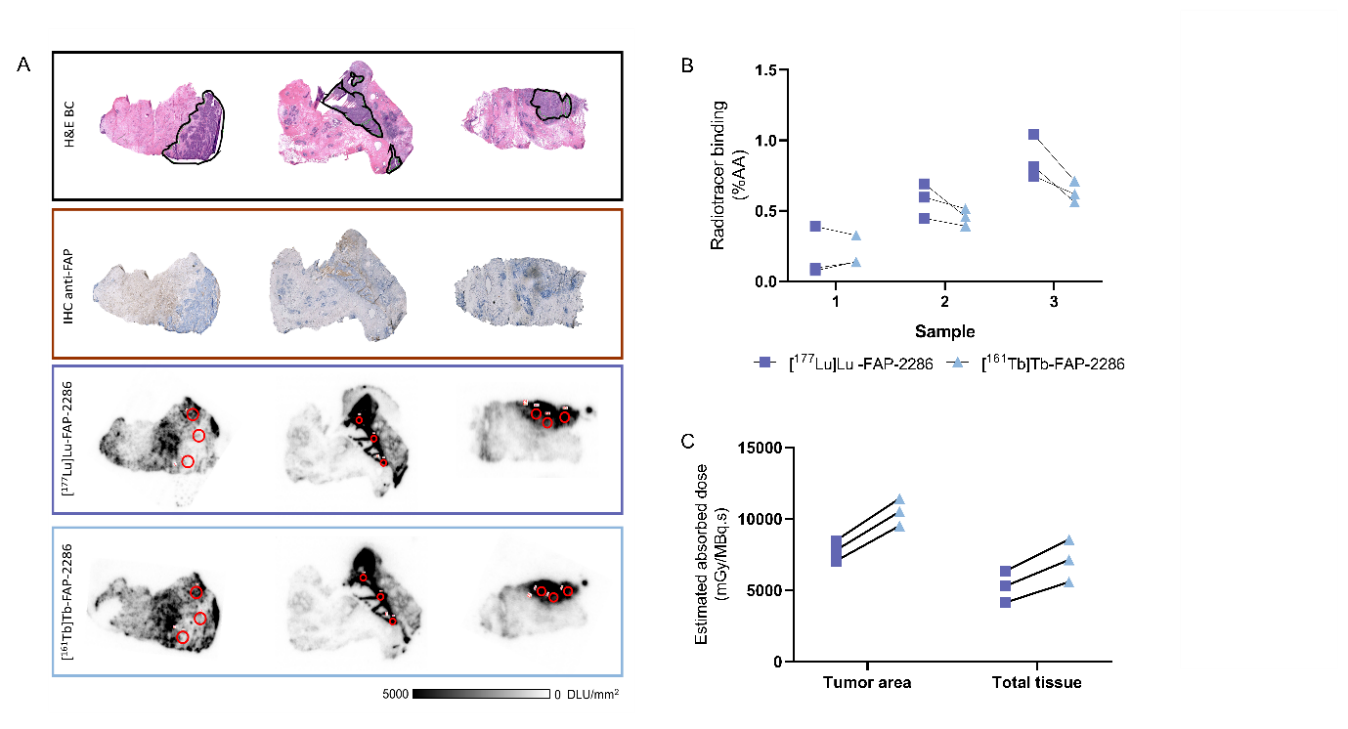


c

b

a

*Supplemental Fig. S11. In vitro autoradiography on patient PDAC samples.* (a) H&E staining of PDAC patient material with tumor delineated in black (top panel), FAP-IHC (second panel) and the autoradiography performed using [^177^Lu]Lu-FAP-2286 and [^161^Tb]Tb-FAP-2286 (lower panels). (b) Quantification of radiopharmaceutical binding in three indicated areas (red circles) demonstrating no significant differences between radiopharmaceutical binding determined by 2-way ANOVA. (c) Calculated absorbed dose to the tumor area and to the total tissue slice in mGy/MBq.s calculated by Monte Carlo simulations.

**References**

1. van der Heide. C.D. et al. *In vitro and ex vivo evaluation of preclinical models for FAP-targeted theranostics: differences and relevance for radiotracer evaluation.* EJNMMI Res. 2024. **14**(1): p. 125.
